# Deubiquitinating enzyme amino acid profiling reveals a class of ubiquitin esterases

**DOI:** 10.1073/pnas.2006947118

**Published:** 2021-01-21

**Authors:** Virginia De Cesare, Daniel Carbajo Lopez, Peter D. Mabbitt, Adam J. Fletcher, Mathieu Soetens, Odetta Antico, Nicola T. Wood, Satpal Virdee

**Affiliations:** ^a^Medical Research Council Protein Phosphorylation and Ubiquitylation Unit, University of Dundee, DD1 5EH, Scotland, United Kingdom

**Keywords:** ubiquitin, DUBs, non-lysine ubiquitination

## Abstract

The reversibility of ubiquitination by the action of deubiquitinating enzymes (DUBs) serves as an important regulatory layer within the ubiquitin system. Approximately 100 DUBs are encoded by the human genome, and many have been implicated with pathologies, including neurodegeneration and cancer. Non-lysine ubiquitination is chemically distinct, and its physiological importance is emerging. Here, we couple chemically and chemoenzymatically synthesized ubiquitinated lysine and threonine model substrates to a mass spectrometry-based DUB assay. Using this platform, we profile two-thirds of known catalytically active DUBs for threonine esterase and lysine isopeptidase activity and find that most DUBs demonstrate dual selectivity. However, with two anomalous exceptions, the ovarian tumor domain DUB class demonstrates specific (iso)peptidase activity. Strikingly, we find the Machado–Joseph disease (MJD) class to be unappreciated non-lysine DUBs with highly specific ubiquitin esterase activity rivaling the efficiency of the most active isopeptidases. Esterase activity is dependent on the canonical catalytic triad, but proximal hydrophobic residues appear to be general determinants of non-lysine activity. Our findings also suggest that ubiquitin esters have appreciable cellular stability and that non-lysine ubiquitination is an integral component of the ubiquitin system. Its regulatory sophistication is likely to rival that of canonical ubiquitination.

## Significance

Ubiquitination involves the covalent attachment of the protein ubiquitin to substrates. It can be reversed by the action of deubiquitinating enzymes (DUBs), thereby providing an important layer of regulation. Originally believed to be restricted to lysine residues, it is emerging that additional amino acids, including serine, threonine and cysteine, are also modified. It remains unknown which DUBs might target these unusual sites for deubiquitination. Herein, we develop representative model substrates and screen 53 DUBs for non-lysine activity, thereby providing important insights into DUB function. Strikingly, we find that a poorly studied DUB class has potent and highly selective serine/threonine activity. These findings suggest that non-lysine ubiquitination rivals the regulatory sophistication of its conventional counterpart and might serve distinct cellular functions.

Ubiquitination impacts on almost all cellular processes and is carried out by a multienzyme cascade involving E1 activating enzymes (E1s), E2 conjugating enzymes (E2s), and E3 ligases (E3s) (1, 2). E3s confer substrate specificity and can broadly be classified into two main classes. The largest class consists of RING E3s which use an adapter-like mechanism to facilitate direct transfer of ubiquitin (Ub) from thioester-linked E2 (E2∼Ub) to substrate (3). On the other hand, engagement of E2∼Ub by HECT-like E3s results in formation of a thioester-linked E3 intermediate that carries out substrate transfer autonomously (4, 5). Conventionally, Ub is linked to the ε-amino group of lysine side chains by an isopeptide bond, or, less frequently, it can be appended to the α-amino group of proteins via a regular peptide bond (1). Multiple residues within Ub itself can also become ubiquitinated, allowing the formation of Ub polymers with distinct linkage topologies that can mediate different cellular processes (6). Ubiquitination is a dynamic modification and is reversed by the action of deubiquitinating enzymes (DUBs). Approximately 100 DUBs have been identified in humans and are assigned to seven distinct classes (7). For the majority of DUBs, substrate specificity is poorly understood, with most biochemical insights gained thus far coming from studies toward isopeptide-linked Ub polymers. Alterations in substrate ubiquitination are often the molecular basis for pathology, and DUBs have become attractive therapeutic targets (7).

Although ubiquitination is typically a lysine-specific posttranslational modification, the RING E3 MIR1 encoded by Kaposi’s sarcoma associated-herpes virus can evade host immune responses by carrying out ubiquitination of cysteine within major histocompatibility complex class I (MHC-I) molecules (8, 9). This promotes their endocytosis and lysosomal degradation. It was subsequently shown that MIR1 from murine γ-herpes virus also ubiquitinates MHC-I molecules but targets serine, threonine, and lysine residues and promotes their degradation by endoplasmic reticulum-associated degradation (ERAD) (10). However, the adapter-like mechanism demonstrated by RING E3s relies upon the active site of the E2 to mediate transfer chemistry. This grants E2s with the important ability to direct ubiquitination to specific sites within a substrate (1, 11). It was subsequently demonstrated that murine MIR1 functions with the poorly studied mammalian E2 UBE2J2, which was shown to possess cellular serine/threonine esterification activity (12).

In further support of the physiological importance of non-lysine ubiquitination, HECT-like E3s also possess intrinsic esterification activity. MYCBP2/Phr1 has important roles in neural development and programmed axon degeneration (13) and has highly selective threonine esterification activity (5). Furthermore, the E3 HOIL-1 has fundamental roles in immune signaling (14) and forms ester linkages with serine/threonine residues within Ub polymers and protein substrates (15). However, the cellular stability of Ub esters is unknown so it is not clear whether ubiquitination of hydroxy amino acids can serve as a sustained cellular signal or if it is restricted to transient roles.

The emerging evidence that non-lysine ubiquitination has important roles across a range of fundamental cellular process, such as viral infection, ERAD, axon degeneration, and immune signaling, places urgent emphasis on establishing which of the ∼100 DUBs might confer Ub esterase activity and serve as negative regulators of this distinct form of ubiquitination. A small panel of DUBs have been tested for activity against an ester-linked substrate which indicated that certain DUBs do possess esterase activity and this need not be mutually exclusive with isopeptidase activity (16). However, comprehensive, DUB profiling, across multiple classes, remains to be carried out.

Here, we synthesize model substrates consisting of threonine/serine that are ester-linked to Ub. Using a high-throughput matrix-assisted laser desorption/ionization time-of-flight (MALDI-TOF) DUB assay (17), we profile two-thirds of known active Ub DUBs for selectivity toward linkage chemistry (lysine isopeptide versus threonine ester). Our findings show that the vast majority of DUBs demonstrate isopeptidase and esterase activity with comparable kinetics. Isopeptidase versus esterase activity is largely inherent to DUB class as ubiquitin-specific protease (USP) and ubiquitin C-terminal hydrolase (UCH) DUBs displayed little preference for linkage chemistry whereas ovarian tumor domain (OTU) DUBs were largely dedicated isopeptidases. Two exceptions were TRABID and the virally encoded DUB, vOTU. Strikingly, the Machado–Joseph disease (MJD) class demonstrated selective threonine and serine esterase activity. We show that esterase selectivity is maintained toward model peptide substrates. Importantly, we also establish that Ub esters can have an intracellular half-life of at least 1 h. We also demonstrate that, in vitro*,* the E2 UBE2J2 possesses selective esterification activity, as inferred by its auto modification profile, which is specifically reversed by the MJD member JOSD1. Using chemically synthesized fluorescent substrates, we quantify the catalytic efficiency of JOSD1 and find it to be a highly efficient Ub esterase (*k*_*cat*_*/K*_*M*_ = 3.5 × 10^4^ M^−1^⋅s^−1^), an efficiency comparable to that of the most efficient isopeptidases. While we could only quantify catalytic efficiency for threonine esterase activity, complementary assays suggest JOSD1 serine esterase activity is considerably higher. Taken together, our findings further support the biological significance of non-lysine ubiquitination and demonstrate that its regulatory sophistication is comparable to that of canonical ubiquitination. The complementary activity profiles of certain OTU DUBs with that of JOSD1 might also allow them to be used as research tools for dissecting the emerging prevalence of non-lysine substrate ubiquitination. Our ester-linked model substrates should also facilitate the development of robust assays for inhibitor screening against MJD members.

## Results

### DUB Esterase and Isopeptidase Activity Profiling.

**Fig. 1.**
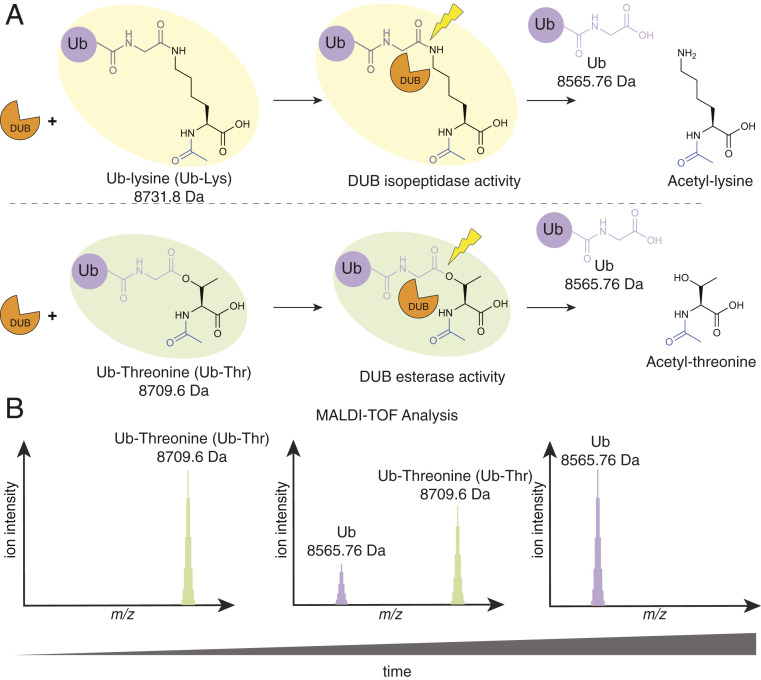
MALDI-TOF–based methodology for profiling DUB isopeptidase/esterase activity. (*A*) DUBs are incubated either with ubiquitin-lysine (Ub-Lys) or ubiquitin-threonine (Ub-Thr). (*B*) Reactions are quenched by addition of TFA (2%), spotted on an AnchorChip 1536 target and analyzed by MALDI-TOF mass spectrometry. DUB isopeptidase or esterase activity releases free lysine or threonine and generates native Ub (8565.76 Da) in a time-dependent manner. Ion intensity ratios for peaks corresponding to Ub-Lys (8731.8 Da)/Ub-Thr (8709.6 Da) versus Ub (8565.76 Da) are used for qualitative assessment of % cleavage.

To determine DUB activity and specificity toward either ester or isopeptide bonds, we employed a previously developed MALDI-TOF DUB assay (17) and the model substrates Ub-Lysine (Ub-Lys) and Ub-Threonine (Ub-Thr) (Fig. 1 *A* and *B*). However, as ubiquitination site context is typically variable, lysine vs. threonine specificity could be mediated by extended DUB–substrate interactions, which our model substrates would not be able to determine. Consequently, in the context of a DUBs physiological substrate, amino acid specificity might be different to that observed with these model substrates. For similar reasons, these substrates would not recapitulate the diverse architectures of polyUb species and would not be able to evaluate DUB-mediated exo- or endo-cleavage (7). Ub-Lys was chemically prepared using a modified implementation of genetically encoded orthogonal protection and activated ligation (GOPAL) technology (18) (*SI Appendix*, Fig. S1). Ub-Thr was chemoenzymatically prepared using a reconstituted E1-E2-E3 cascade based on the RING-Cys-Relay E3 machinery from MYCBP2 (5) (*SI Appendix*, Fig. S2). For both amino acid substrates, the α-amino group was acetylated, which helped mirror the peptide context the model substrates were reflective of, and also prevented potential O-N acyl transfer of Ub-Thr to a peptide-linked species (19).

**Fig. 2.**
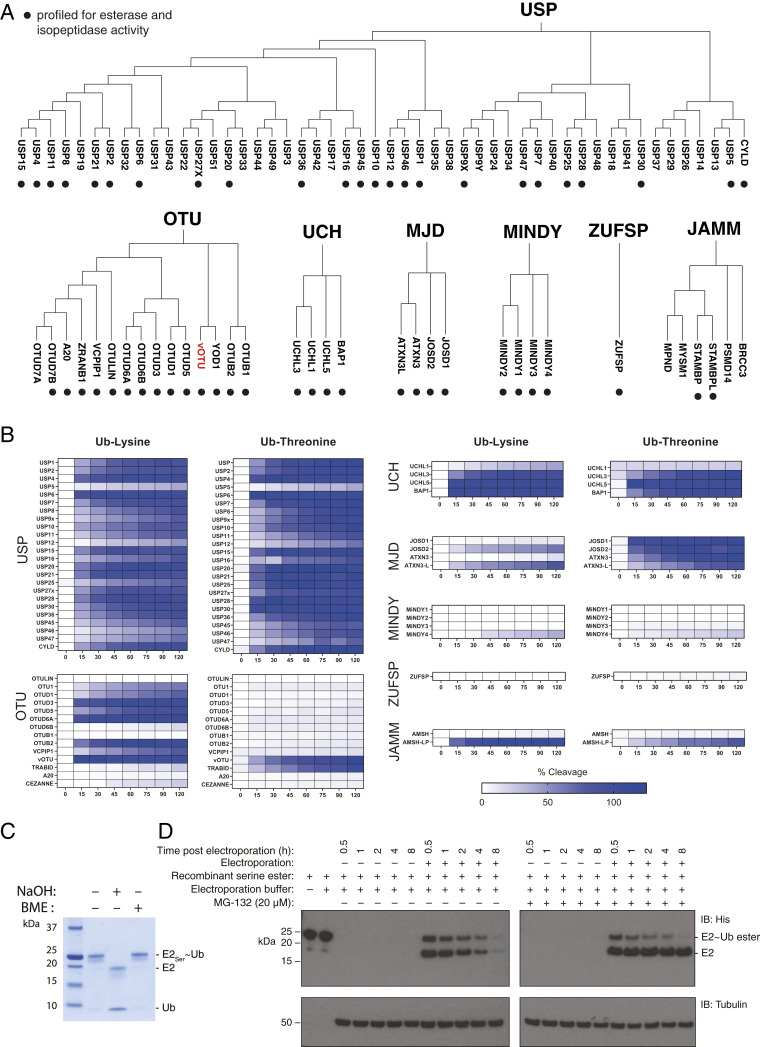
DUB esterase and isopeptidase screen by MALDI-TOF mass spectrometry and assessment of cellular ubiquitin ester stability. (*A*) Phylogenetic classification of deubiquitinating enzymes based on their catalytic domains. Only DUBs that are active and recognize ubiquitin are displayed. The vOTU (red) catalytic domain exists within Protein L, which is encoded by Crimean–Congo hemorrhagic fever virus. DUBs annotated with a solid circle correspond to those profiled for activity in this study. (*B*) A panel of 53 DUBs were tested for their activity toward model substrates Ub-Lys and Ub-Thr. Reactions were then quenched by addition of TFA (2%) at the relevant time points and spotted onto a 1536 AnchorChip target plate, followed by MALDI-TOF analysis (17). Results are reported as percent of cleavage in a scale from white (no activity) to dark blue (100% substrate consumption). Employed DUB concentrations are specified in *SI Appendix*, Table S1. (*C*) An engineered E2∼Ub conjugate linked to the Ub C terminus via a serine ester (E2_Ser_∼Ub) was used as model ester-linked substrate. Resistance to BME, but sensitivity to NaOH, is consistent with the conjugate being ester-linked. (*D*) E2_Ser_∼Ub was delivered into HEK293 cells by electroporation and then transferred directly into prewarmed media and then incubated at 37 °C, 5% CO_2_. Samples were taken at the indicated time points postelectroporation. The experiment was replicated in parallel, using HEK293 pretreated with 20 μM MG132 for 30 min, and cells incubated in media containing MG-132 throughout.

We screened a panel of 53 recombinant DUBs belonging to all seven known DUB families (Fig. 2 *A* and *B*) (7): USP, OTU, UCH, JAB1/MPN/Mov34 metalloenzyme (JAMM), MJD, motif interacting with Ub-containing novel DUB family (MINDY), and zinc finger with UFM1-specific peptidase domain protein (ZUFSP). DUBs were incubated with Ub-Lys and Ub-Thr, and, as positive controls, they were also incubated with an alternative Ub-derived substrate (either an isopeptide-linked diUb or Ub with a C-terminal peptide-linked adduct) known to be processed by the DUB under investigation (*SI Appendix*, Fig. S3 and Table S1). However, for the DUBs JOSD1, OTU1, OTUD6A, and OTUD6B, a readily accessible substrate which is cleaved hitherto remains to be identified. For quantification and normalization purposes, the ratio of the area of the substrate ion intensity signal (Ub-Lys or Ub-Thr) and the area of the product signal (Ub) were recorded and extrapolated to a standard curve, based on defined substrate/product ratios, enabling calculation of percent substrate cleavage (*SI Appendix*, Fig. S4). By comparing the percent of cleavage of Ub-Lys versus Ub-Thr as a function of time, we determined activity and specificity toward the two model substrates (Fig. 2*B*). In practice, fractional cleavage was sometimes found to exceed 100%. This is because DUB, which is absent when producing the standard curve, can suppress the mass spectrometry (MS) signal of low abundance Ub species (e.g., Ub-Lys or Ub-Thr after appreciable cleavage), and data were not normalized for this phenomenon.

### Esterase and Isopeptidase Selectivity Is Largely Inherent to DUB Classification.

We find that DUBs belonging to the USP and UCH family in general cleave both Ub-Lys and Ub-Thr substrates with comparable kinetics (Fig. 2*B*). The USPs are the largest class, consisting of ∼60 members, whereas UCHs are a smaller classification, consisting of only 4 members (7). The only USPs with negligible activity toward either substrate are USP5 and USP12. However, lack of USP5 activity is anticipated as it is only functional toward polyUb with an intact free Ub C terminus (20). In the case of the UCH class, UCHL1 is found to only cleave the isopeptide-linked substrate while UCHL3, UCHL5, and BAP1 show no appreciable preference between the two model substrates (Fig. 2*B*).

In contrast to USP and UCH classes, OTU family members invariably demonstrate efficient lysine isopeptidase activity toward our model substrate, but negligible threonine esterase activity (Fig. 2*B*). Two notable exceptions are vOTU and TRABID. In the context of this assay, TRABID demonstrates selective threonine esterase activity whereas vOTU demonstrates robust activity toward both substrates (Fig. 2*B*). The DUB vOTU is encoded by the deadly human pathogen, Crimean Congo hemorrhagic fever virus. In addition to its ability to hydrolyze four out of six tested isopeptide-linked Ub polymer types (21), it has also been shown to have relaxed substrate scope as it removes the ubiquitin-like modifier ISG15 (21). Thus, the observation that vOTU demonstrates high isopeptidase and threonine esterase activity implies that relaxation of its substrate scope extends to Ub linkage chemistry and that non-lysine ubiquitination may promote mammalian antiviral responses more broadly.

TRABID has been implicated with Wnt and immune signaling (22, 23) and has efficient isopeptidase activity in the context of Lys29- and Lys33-linked Ub polymers (18, 24). However, in our assay, TRABID has negligible isopeptidase activity toward Ub-Lys, consistent with that observed for other, albeit peptide-linked, small molecule substrates (25). Thus, our observation that TRABID has high activity toward Ub-Thr implies that its esterase activity is more promiscuous than its isopeptidase activity, and hence a significant proportion of its physiological substrates may in fact be non-lysine ubiquitination sites.

The MJD family is a small class of DUBs consisting of four members (26). Unlike the other DUB classifications which are found in all eukaryotes, MJD DUBs are absent in yeast and might be reflective of a specific demand of higher eukaryotes. Strikingly, with the exception of ATXN3L, all MJD DUBs demonstrate preferential threonine esterase activity (Fig. 2*B*). This is particularly notable for JOSD1 where isopeptidase activity is negligible but quantitative cleavage of the Ub-Thr substrate is observed after the first time point. Similarly, Josephin-2 (JOSD2) cleaves both substrates but has a significant preference for the Ub-Thr substrate over the lysine counterpart. ATXN3 also cleaves Ub-Thr more efficiently than Ub-Lys whereas ATXN3L does not demonstrate any notable substrate preference (Fig. 2*B*).

We also tested the recently discovered MINDY and ZUFSP classes of DUB. The MINDY class consists of four members, which demonstrate *exo* (cleaving from the distal end) activity toward extended Lys48 linked Ub polymers (27). ZUFSP consists of a single founding member (ZUFSP/ZUP1) and is specific for Lys63-linked Ub polymers and is involved in DNA repair (28–31). Consistent with activity of these DUBs being dependent on polyUb linkage context, negligible activity was observed toward either of our model isopeptide or ester-linked substrates (Fig. 2*B*).

Unlike the other DUB classes identified thus far which are cysteine isopeptidases/peptidases, the JAMM class of DUBs are metalloproteases (26). Two of the six functional JAMM class DUBs included in our panel are AMSH and AMSH-LP, which have both been shown to have specific activity toward isopeptide-linked Lys63 Ub polymers with comparable efficiency. Interestingly, under the enzyme concentrations employed, AMSH displays no detectable esterase nor isopeptidase activity toward the model substrates whereas AMSH-LP is active against both model substrates (Fig. 2*B*) (32).

### Cellular Stability of Ubiquitin Esters.

The cellular stability of a Ub ester would determine whether posttranslational modification via this linkage would be restricted to transient roles or whether it might also serve as a sustained cellular signal. Intrinsic susceptibility to hydrolysis and the action of the highly efficient and promiscuous esterase activity found in mammalian cells might limit its cellular function to transient roles (33). To assess the cellular stability of a serine residue esterified with Ub, we devised an experiment. Here, we prepared an engineered E2∼Ub conjugate (E2_Ser_∼Ub), linked via a serine ester (Fig. 2*C*), and delivered it into cells via electroporation (Fig. 2*D*) (34, 35). Strikingly, we found that the conjugate had a cellular half-life of 1 to 2 h and could still be detected after 8 h, thereby suggesting that ubiquitination of hydroxy amino acids can serve as a sustained cellular signal.

### Validation of Selective USP and OTU Isopeptidase Activity.

**Fig. 3.**
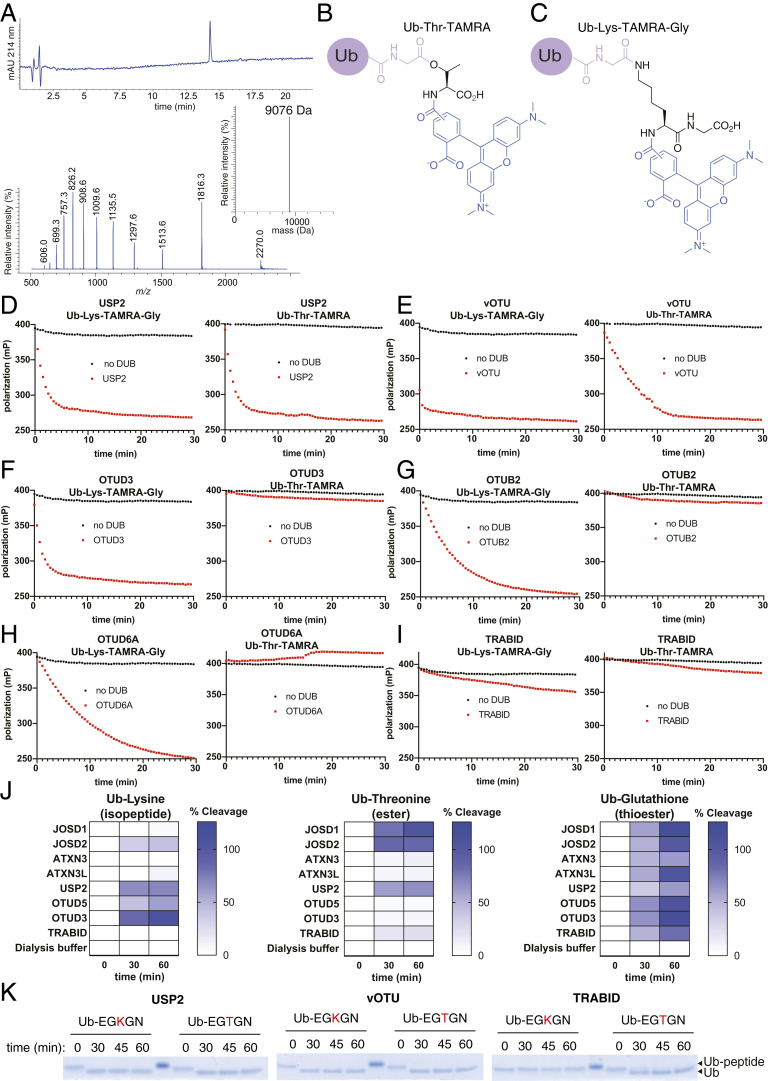
Validation of selected DUB activity toward Ub-Lys-TAMRA-Gly and Ub-Thr-TAMRA by continuous FP-based assay, and ubiquitinated model lysine and threonine peptides by gel-shift assay. (*A*) LC-MS characterization data for Ub-Thr-TAMRA: HPLC chromatogram for purified Ub-Thr-TAMRA monitoring at 214 nm, electrospray ionization mass spectrum for Ub-Thr-TAMRA and deconvoluted mass spectrum for Ub-Thr-TAMRA (theoretical mass = 9078.65; observed mass = 9076 Da). (*B*) Chemical structure of the fluorescent Ub-Thr-TAMRA. (*C*) Chemical structure of commercial Ub-Lys-TAMRA-Gly. (*D*) Consistent with the MALDI-TOF data, USP2 (0.125 μM) demonstrates comparable lysine isopeptidase and threonine esterase activity. (*E*) The virally encoded DUB, vOTU (1.5 μM), has both lysine isopeptidase and threonine esterase activity with an apparent selectivity for the isopeptide-linked model substrate. (*F*–*H*) OTUD3, OTUB2, and OTUD6A (1.5 μM) demonstrate selective lysine isopeptidase activity. (*I*) Inconsistent with the MALDI-TOF data using the simple model substrates Ub-Lys and Ub-Thr, TRABID (1.5 μM) demonstrates both weak isopeptidase and esterase activity. (*J*) A subset of DUBs were buffer exchanged by dialysis to remove DTT. To establish whether Ub-Glutathione cleavage was DUB-mediated, or due to thiolysis by residual DTT, the buffer the samples were dialyzed against was added as a control. (*K*) To ascertain whether the observed DUB activity profiles were observed in a peptide context, activity was assessed toward unlabeled ubiquitinated model peptides (Ub-EGKGN and Ub-EGTGN). Consistent with both MALDI-TOF data using unlabeled amino acid substrates and FP data using TAMRA-labeled substrates, both USP2 and vOTU (0.75 μM) demonstrate robust isopeptidase and esterase activity. Consistent with the MALDI-TOF data toward unlabeled Ub-Lys and Ub-Thr, TRABID (0.75 μM) demonstrates negligible lysine isopeptidase activity, but robust threonine esterase activity. For clarity, the ubiquitinated amino acids in the peptides are highlighted in red.

To validate the activity profiles determined by the MALDI-TOF assay format, we initially prepared a fluorescent model substrate where the α-amino group of threonine is labeled with 5/6-carboxytetramethylrhodamine (TAMRA) (Ub-Thr-TAMRA) (Fig. 3 *A* and *B*). The fluorescent amino acid was linked to Ub via an ester bond using the chemoenzymatic strategy adopted earlier. For comparison, we used commercially available isopeptide-linked Ub-Lys-TAMRA-Gly (Fig. 3*C*). Esterase or isopeptidase activity, respectively, would cleave the fluorescent amino acid from Ub, allowing continuous and quantitative measurement of DUB activity by fluorescence polarization (FP) (36). For orthogonal testing in the FP assay, we selected the USP class DUB USP2 and vOTU, both of which demonstrate high isopeptidase and esterase activity (Fig. 2*B*). We also selected OTUB2, OTUD3, and OTUD6A, which demonstrate isopeptidase specificity, and TRABID, which demonstrates selective threonine esterase activity, despite reports of it being an efficient isopeptidase (Fig. 2*B*). Under these alternative assay conditions, USP2 maintains dual specificity, with comparable observed rates for isopeptidase and threonine esterase activity (0.18 min^−1^ and 0.13 min^−1^, respectively) (Fig. 3*D*). The DUB vOTU also maintains dual specificity, albeit with a preference for Ub-Lys-TAMRA-Gly (Fig. 3*E*). Again, consistent with the MALDI-TOF assay, the OTU DUBs OTUD3, OTUB2, and OTUD6A exhibit isopeptidase specificity as no threonine esterase activity is detected (Fig. 3 *F*–*H*). However, despite demonstrating threonine esterase activity toward the small acetylated threonine substrate, TRABID is only weakly active against Ub-Thr-TAMRA and, unexpectedly, similarly so toward the Ub-Lys-TAMRA-Gly (Fig. 3*I*). To ascertain if the C-terminal glycine residue in the isopeptide-linked substrate might be affecting the ability to process the isopeptide-linked substrate, relative to the less hindered Ub-Thr-TAMRA substrate, we also prepared an analogous Ub-Lys-TAMRA substrate (*SI Appendix*, Fig. S5) and found activity to be comparable toward both (*SI Appendix*, Fig. S6). This is indicative of the TAMRA fluorophore present in Ub-Thr-TAMRA interfering with recognition by TRABID.

### Thioesterase Activity Is Possessed by DUBs with Isopeptidase and Esterase Activity.

It has been reported that ubiquitination can also extend to cysteine residues where the linkage chemistry is a thioester bond (9, 37). It remains unknown if DUBs can also cleave this linkage chemistry. We attempted chemoenzymatic preparation of simple model cysteine substrates (38), analogous to those described above (Figs. 1 and 3), but found them to be unstable by mass spectrometry as they would seemingly rearrange to the peptide-linked cysteine adduct with concomitant loss of the acetyl or TAMRA groups, presumably promoted by the free cysteine carboxylate. We therefore sought an alternative model cysteine-containing substrate and opted for reduced glutathione. Ubiquitinated glutathione (Ub-Glutathione) was prepared using a modification of a reported chemoenzymatic procedure (38) (*SI Appendix*, Fig. S7). As expected, Ub-Glutathione could be purified and was sufficiently stable for DUB activity analysis. However, as our DUB preparations contained dithiothreitol (DTT) reducing agent which would lead to thioester thiolysis, we buffer-exchanged a subset of DUBs with distinct specificity profiles into phosphate-buffered saline (PBS) containing the phosphine-based reducing agent Tris(2-carboxyethyl)phosphine (TCEP). This DUB subset was then tested for cysteine thioesterase activity using the MALDI-TOF assay (Fig. 3*J*). Although the dialysis procedure compromised DUB activity, all DUBs demonstrated Ub-Glutathione thioesterase activity similar to or greater than that toward their preferred isopeptide or ester substrate (Fig. 3*J*).

### TRABID Demonstrates Selective Threonine Esterase Activity toward a Peptide Substrate.

The unexpected esterase activity toward Ub-Thr, and the inconsistent results obtained from this substrate and Ub-Thr-TAMRA, prompted us to prepare alternative substrates based on model peptides (Ac-EGXGN-NH_2_ where X = K or T) that were either isopeptide-linked or ester-linked to Ub. DUB activity toward these peptide substrates would be more reflective of a native protein substrate and could be assessed by electrophoretic shift upon sodium dodecyl sulfate–polyacrylamide gel electrophoresis (SDS-PAGE) analysis. Ubiquitinated peptides were prepared using a reconstituted E1-E2-E3 cascade based on a constitutively active RING E3 (RNF4) (39), or MYCBP2, and were purified by reversed-phase high-performance liquid chromatography (RP-HPLC) (*SI Appendix*, Figs. S8 and S9). We initially tested USP2 and vOTU. Consistent with the activity profiles toward the Ub-Lys and Ub-Thr substrates, USP2 and vOTU cleaved both the isopeptide-linked and threonine-linked peptide substrates within our first time point, supporting the notion that the bispecific isopeptidase and esterase activity demonstrated by the vast majority of USP DUBs and vOTU would extend to protein substrates (Fig. 3*K*).

Consistent with the initial MALDI-TOF assay data using nonfluorophore-labeled amino acid substrates, TRABID demonstrated threonine esterase activity within the peptide context but no lysine isopeptidase activity (Fig. 3*K*). This suggests that, while TRABID has efficient isopeptidase activity toward distinct polyUb linkages (18), it reinforces our earlier assertion that its esterase activity is indeed more promiscuous and TRABID is likely to have unappreciated substrates that are ester-linked to Ub.

### MJD DUBs Have Selective Ubiquitin Esterase Activity.

**Fig. 4.**
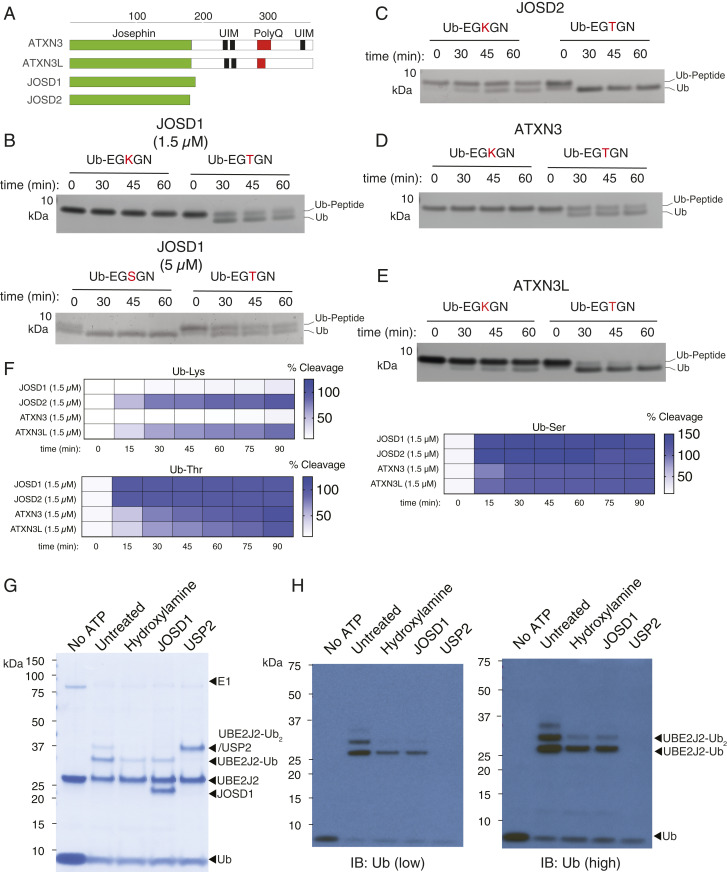
Comprehensive assessment of MJD DUB selective esterase activity toward peptides and the non-lysine automodification product of the E2 conjugating enzyme UBE2J2. (*A*) Domain architecture of MJD DUBs. (*B*, *Top*) Assessment of JOSD1 (1.5 μM) selective threonine esterase activity toward Ub-EGKGN and Ub-EGTGN model peptide substrates. (*B*, *Bottom*) Comparison of JOSD1 (5 μM) activity toward Ub-EGSGN and Ub-EGTGN. (*C*) Assessment of JOSD2 (1.5 μM) selective threonine esterase activity toward model peptide substrates. (*D*) Assessment of ATXN3 (1.5 μM) selective threonine esterase activity toward model peptide substrates. (*E*) Assessment of ATXN3L (1.5 μM) selective threonine esterase activity toward model peptide substrates. Gels presented in *B*–*E* were visualized by silver staining. (*F*) MALDI-TOF assay data demonstrating that, with the exception of ATXN3L, all MJD family members demonstrate selective threonine/serine esterase activity toward model Ub-Lys, Ub-Thr, and Ub-Ser substrates. (*G*) Enzymatic reconstitution in PBS (pH 7.5) with UBE2J2 (20 μM), E1 (200 nM), Ub (50 μM), and ATP (2 mM). Reaction was incubated for 30 min at 37 °C, and E2 loading was terminated by addition of Compound 1 E1 inhibitor (25 μM) (55). Samples were then treated with buffer control or DUB (3 μM) for 30 min at 30 °C. Reactions were terminated with SDS loading buffer containing BME or BME plus hydroxylamine (0.5 M) and incubated for 30 min at 37 °C prior to SDS-PAGE analysis. (*H*) High and low exposures for anti-Ub immunoblot (IB) of samples analyzed in *G*.

The Machado–Josephin family (also referred to as Josephins) are a small class of DUBs consisting of four members (ATXN3, ATXN3L, JOSD1, and JOSD2). The founding and most-studied member is Ataxin-3 (ATXN3) (40, 41). This protein is encoded by the gene responsible for the neurological condition spinocerebellar ataxia type-3, or Machado–Joseph disease, from which the MJD class takes its name. Machado–Joseph disease is an autosomal dominant neurodegenerative disorder caused by polyQ tract expansion (42). All MJD proteins share a common globular catalytic cysteine protease domain of ∼180 amino acids, known as the Josephin domain. ATXN3 and ATXN3L sequences consist of the Josephin domain and a disordered C-terminal tail where the polyQ tract and two or three ubiquitin-interacting motifs (UIMs) are located. The latter bind polyUb chains (Fig. 4*A*) (41, 43). JOSD1 and JOSD2, on the other hand, consist of little more than the catalytic Josephin domain.

JOSD1 is implicated with membrane dynamics (44), cancer chemoresistance (45), and antiviral response (46), yet its physiological substrates are poorly defined (45, 46). It has been reported that JOSD1 and ATXN3 can exist as monoubiquitinated forms which have a modest activating effect on their ability to cleave polyUb species (44, 47). Importantly, it has recently been shown that elevated JOSD1 levels are found in gynecological cancers and this correlates with poor prognosis (45). JOSD1 also promotes chemoresistance by stabilizing antiapoptotic myeloid cell leukemia 1 (MCL-1). We too observe a high molecular weight form of JOSD1 when transiently overexpressed in HEK293 cells (*SI Appendix*, Fig. S10). However, this was neither sensitive to USP2 nor hydroxylamine treatment (*SI Appendix*, Fig. S11). We could also immunologically detect JOSD1 in various mouse tissues, with highest levels of expression observed in the heart, liver, kidney, and spleen in mouse tissue lysates (*SI Appendix*, Fig. S10). Although MJD DUBs have been shown to have isopeptidase activity toward polyUb substrates, this has not been quantified, and the qualitative data that do exist involve high micromolar concentrations of DUB and/or lengthy incubation times (e.g., 16 to 20 h) (44). To reconcile the sluggish isopeptidase kinetics, it has been proposed that ATXN3 may serve as a cellular timer (48). Another possibility is that the precise nature of the physiological substrates is yet to be determined.

Our observation that MJD DUBs have potent threonine esterase activity raised the possibility they were an unappreciated class of specific Ub esterase. To explore this possibility further, we tested the four MJD DUBs for activity against the peptide substrates. We also managed to prepare limiting amounts of a substrate based on a serine peptide (Ac-EGSGN-NH_2_) using the reconstituted MYCBP2 system (*SI Appendix*, Fig. S12). JOSD1 demonstrated highly specific threonine esterase activity in this context (Fig. 4*B*). Strikingly, JOSD1 serine esterase activity was found to be considerably higher than that toward threonine (100% cleavage after first time point) (Fig. 4*B*). JOSD2 completely cleaved the threonine substrate within the first time point but also demonstrated some lysine isopeptidase activity (Fig. 4*C*). ATXN3 demonstrated specific threonine esterase activity whereas, for ATXN3L, isopeptidase activity was also discernible (Fig. 4 *D* and *E*). These observations are distinct from those observed with the MALDI-TOF screen employing Ub-Lys and Ub-Thr substrates where JOSD2 and ATXN3L exhibited modest or negligible selectivity, respectively. To confirm this was attributable to the different substrates employed, we reassessed JOSD2 and ATXN3L activity toward Ub-Lys and Ub-Thr using a complementary electrospray mass spectrometry assay (*SI Appendix*, Fig. S13) (5). Here, we found that the selectivity profile is similar to that observed with the MALDI-TOF assay, verifying that the relaxed specificity is due to substrate context.

We also found that ATXN3 demonstrates considerably higher esterase activity toward the serine peptide compared to the threonine peptide (*SI Appendix*, Fig. S14). These findings imply that all four MJD DUBs have highly selective, if not specific, esterase activity toward our degenerate peptide substrates. Unfortunately, we could not prepare sufficient quantities of serine peptide substrate to test all four MJD members so it remains a possibility that JOSD2 and ATXN3L also demonstrate greater serine esterase activity in a peptide context.

To assess whether, in principle, JOSD2 and ATXN3L also have serine esterase activity, we chemoenzymatically prepared the simple ubiquitinated serine substrate (Ub-Ser) in sufficient yield for comparison of all MJD members using the MALDI-TOF assay (*SI Appendix*, Fig. S15). Under the employed assay conditions, the MJD class demonstrated both serine and threonine esterase activity with comparable efficiency under the employed conditions (Fig. 4*F*).

We next assessed whether JOSD1 esterase activity and specificity were maintained in a protein context. The lack of tools for studying non-lysine ubiquitination precluded the development of assays based on a physiological protein substrate. However, the E2 conjugating enzyme UBE2J2 has been reported as having esterification activity and undergoes auto modification in the presence of E1, Ub, and adenosine 5′-triphosphate (ATP) (5, 12). The presence of ester linkages would allow the Ub-modified E2 to be used as a model protein substrate of DUB esterase activity. We therefore tested if the auto modifications were ester-linked (5). Ester-linked conjugates were determined by assessment of which were stable after 2-mercaptoethanol (BME) treatment but were cleaved after combination treatment with BME and hydroxylamine. Conjugates that were cleaved after BME treatment would correspond to those that are thioester-linked whereas those that persisted after combination treatment would correspond to those that are isopeptide-linked. We found that a predominant ester-linked Ub adduct is formed, together with a minor (iso)peptide-linked adduct (Fig. 4 *G* and *H*). Strikingly, when employed as a model substrate for JOSD1, specific and quantitative esterase activity was observed (Fig. 4 *G* and *H*). Furthermore, treatment with USP2 removed major and minor bands, thereby validating our earlier findings that USP2 has both esterase and isopeptidase activity.

### Quantification of JOSD1 Threonine Esterase Activity.

**Fig. 5.**
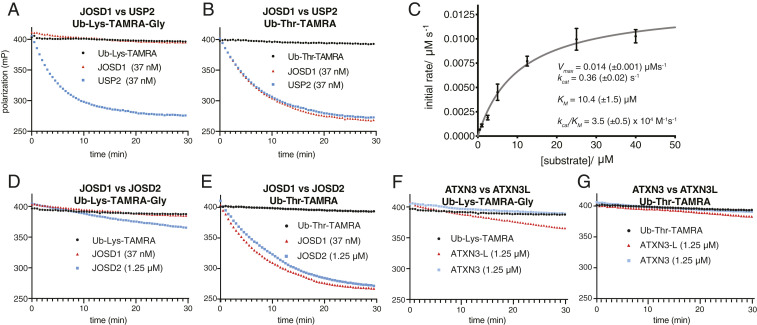
Characterization of MJD DUB esterase activity by FP-based assay toward model amino acid substrates. (*A*) Ub-Lys-TAMRA-Gly tested at 250 nM. The USP class DUB USP2 demonstrates efficient isopeptidase activity toward Ub-Lys-TAMRA-Gly whereas JOSD1 isopeptidase activity is undetectable. (*B*) JOSD1 demonstrates efficient esterase activity toward Ub-Thr-TAMRA with comparable kinetics to that of USP2. (*C*) Steady-state Michaelis–Menten analysis for JOSD1 esterase activity toward Ub-Thr-TAMRA (JOSD1 assay concentration was 37 nM). (*D*) Negligible JOSD2 isopeptidase activity is also observed toward Ub-Lys-TAMRA-Gly (note JOSD2 concentration is 1.25 μM). (*E*) JOSD2 is a less efficient esterase than JOSD1 toward Ub-Thr-TAMRA, but cleavage kinetics are comparable when JOSD2 concentration is increased ∼30-fold relative to JOSD1. (*F*) ATXN3 esterase activity is negligible toward the synthetic Ub-Lys-TAMRA-Gly substrate whereas ATXN3L exhibits weak isopeptidase activity. (*G*) Both ATXN3 and ATXN3L exhibit negligible esterase activity toward Ub-Thr-TAMRA.

We next benchmarked JOSD1 threonine esterase activity against USP2 using the FP assay with the fluorescent Ub-Lys-TAMRA-Gly and Ub-Thr-TAMRA substrates. USP2 is a highly active DUB (*k*_*cat*_/*K*_*M*_ = ∼2.5 × 10^5^ M^−1^⋅s^−1^) (49) and has been used as a research tool to selectively remove Ub from cellular protein substrates by presumed selective isopeptidase activity (50). Consistent with mass spectrometry and gel-based assays, USP2 readily cleaved Ub from Ub-Lys-TAMRA-Gly and Ub-Thr-TAMRA with similar kinetics (Fig. 5 *A* and *B*). However, JOSD1 readily cleaved the Ub-Thr-TAMRA substrate with an observed rate constant comparable to that of USP2 (Fig. 5*B*) but exhibited no detectable isopeptidase activity toward Ub-Lys-TAMRA-Gly (Fig. 5*A*). To determine if the additional glycine residue in Ub-Lys-TAMRA-Gly was affecting isopeptidase activity, we tested all MJD DUBs against our synthesized Ub-Lys-TAMRA substrate and observed equivalent cleavage kinetics (*SI Appendix*, Fig. S6).

To quantify the catalytic efficiency of JOSD1 esterase activity and determine its catalytic parameters, we carried out Michaelis–Menten analysis toward Ub-Thr-TAMRA (Fig. 5*C*). Catalytic turnover [*k*_*cat*_ is 0.36 (±0.02) s^−1^] and the Michaelis constant [*K*_*M*_ is 10.4 (±1.5) μM] are indicative of the observed esterase activity being largely driven by *k*_*cat.*_ The resultant specificity constant [*k*_*cat*_*/K*_*M*_ = 3.5 × 10^4^ M^−1^⋅s^−1^] is comparable to that of USP21 for the K48 Ub dimer (51), which is at the high end of the spectrum of kinetically quantified DUB isopeptidase activity. It should be noted that, on account of JOSD1 esterase activity being considerably higher toward serine than threonine in our peptide-based substrates (Fig. 4*B*), a much higher specificity constant might be found with a serine substrate. Taken together, these findings are consistent with JOSD1 having the potential to mediate dynamic cellular deubiquitination via its esterase activity. JOSD2 also demonstrated selective esterase activity (Fig. 5 *D* and *E*). However, with these TAMRA-labeled substrates ATXN3 and ATXN3L demonstrated negligible or undetectable activity, respectively (Fig. 5 *F* and *G*).

### JOSD1 Esterase Activity Is Mediated by the Canonical Catalytic Site.

**Fig. 6.**
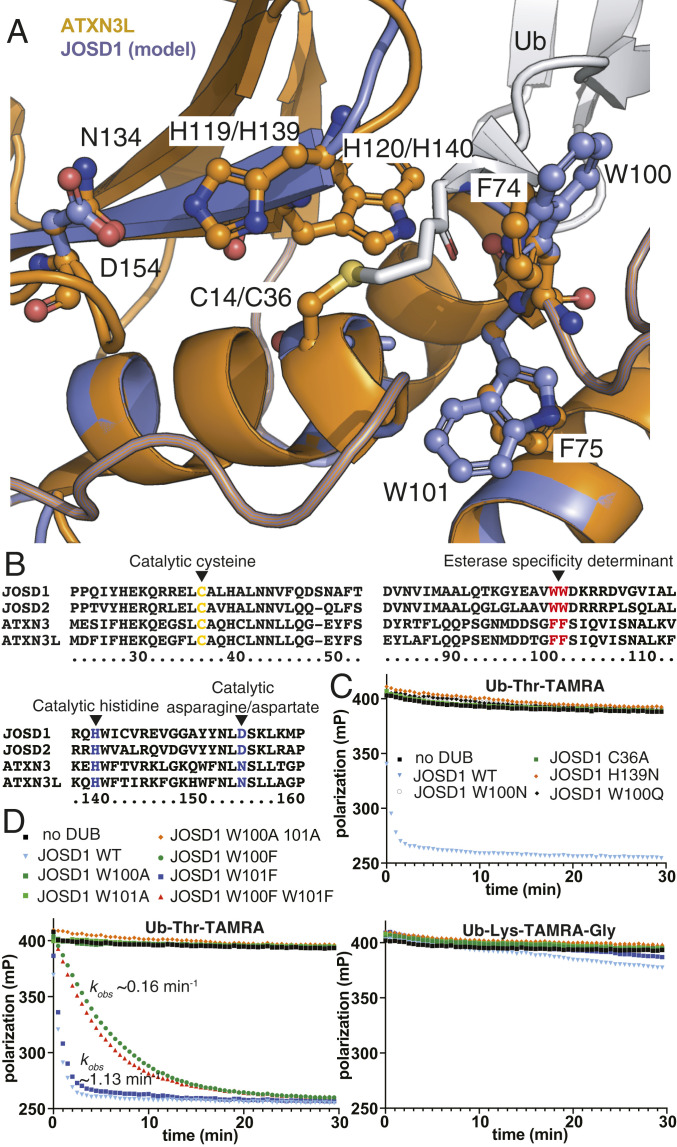
Determinants of MJD DUB esterase selectivity. (*A*) Superposition of the active site for a JOSD1 homology model with that of the ATXN3L:Ub complex (PDB ID code 3O65). (*B*) Sequence alignment for the MJD DUB class. Catalytic residues and those proposed to be important for esterase activity are highlighted. (*C*) FP assay demonstrating that JOSD1 esterase activity is dependent on canonical catalytic residues and the hydrophobic W100 residue. Concentrations of Ub-Thr-TAMRA and JOSD1 variant were 250 nM and 500 nM, respectively. (*D*) Introduction of a W100F mutation into JOSD1 reduced activity by approximately sevenfold whereas a W101F mutant retained WT levels of activity. Mutations had no discernible effect on isopeptidase activity. Concentrations of Ub-Thr-TAMRA/Ub-Lys-TAMRA-Gly and JOSD1 variant were 250 nM and 750 nM, respectively.

The crystal structure of ATXN3, which has been solved in complex with a Ub suicide substrate probe (48), allowed us to build a high confidence homology model (99%) for JOSD1 with Phyre2 (52). In the experimental structure, the active site residues are well resolved, and, although a substrate is absent, residues that would be in immediate proximity of the modified substrate amino acid can be approximated (Fig. 6 *A* and *B*). In light of the unexpected esterase activity, we first determined whether it was dependent on the established catalytic cysteine within JOSD1 (Fig. 6 *A* and *B*). JOSD1 isopeptidase activity has been shown to be dependent on C36 (44). We also found that a C36A mutant of JOSD1 abolishes activity toward Ub-Thr-TAMRA (Fig. 6*C*). Mutation of the histidine belonging to the catalytic triad (H139) also abolishes activity (Fig. 6*C*). These results indicate that the catalytic triad centering on C36 in JOSD1, and presumably the homologous residue in other MJD class DUBs, mediates efficient esterase activity.

### Assessment of the Structural Basis for MJD DUB Esterase Activity.

To determine catalytic residues that might facilitate the selective threonine esterase activity, we studied the experimental ATXN3-Ub structure and its superposition with our JOSD1 homology model (Fig. 6*A*). A conspicuous feature of the ATXN3 and JOSD1 catalytic sites is their hydrophobic nature. The JOSD1 model places two tryptophan residues in the active site whereas ATXN3 has two crystallographically determined phenylalanine residues at the equivalent positions (Fig. 6 *A* and *B*). In some JOSD1 orthologs, the residue equivalent to W101 is also a phenylalanine, raising the possibility that the phenylalanine residue can serve a similar function to the tryptophan (*SI Appendix*, Fig. S16). To test if the hydrophobic residues were contributing to JOSD1 esterase activity, we mutated the tryptophan residues and tested activity. Strikingly, W100Q and a W100N mutation abolished activity, implying that hydrophobic character is important (Fig. 6*C*). W100A and W101A mutations also abolished JOSD1 esterase activity, indicating the aromatic nature, shared by the phenylalanine residue found in JOSD1 orthologs and in ATXN3 and AXTN3L, may also be significant (Fig. 6*D*). We next assessed whether the tryptophan and phenylalanine residues might serve shared functions. We introduced W100F and W101F mutations, either singularly or in combination, into JOSD1 (Fig. 6*D*). In support of a shared function, threonine esterase activity of the W100F mutant was modestly impaired whereas a W101F mutant retained wild-type (WT) levels of activity. To test if the homologous phenylalanine residues in ATXN3 might have similar function, we assayed alanine mutants in the MALDI-TOF and peptide-based gel shift assays (*SI Appendix*, Figs. S16 and S17). While an ATXN3 F74A mutant had no discernible effect on activity, an F75A mutant abolished ATXN3 esterase activity, underscoring the functional relevance of the second hydrophobic residue across the MJD class. We also explored the effect of F74W and F75W mutations on ATXN3 (*SI Appendix*, Fig. S16). No detectable activity was observed for the F74W mutation, indicative of the tryptophan at this position being incompatible in the context of the ATXN3 active site. The F75W mutant retains WT levels of activity, suggestive of the phenylalanine residue in ATXN3 and the tryptophan residue in JOSD1 having similar molecular function.

## Discussion

Non-lysine ubiquitination is emerging as a physiologically important posttranslational modification. As systems-wide technologies for identifying ubiquitination sites are tailored for lysine ubiquitination, the scale of cellular non-lysine ubiquitination remains to be determined. We tested the activity of 53 DUBs against both lysine and non-lysine ubiquitinated model substrates, making the current work the most extensive and cross-validated study on non-lysine DUB activity to date. The results show that isopeptidase versus esterase activity is largely dependent on DUB phylogeny. We found that, on the whole, USP and UCH class DUBs mediate both isopeptidase and esterase activity with comparable kinetics. On the other hand, the OTU DUB classification is largely devoid of esterase activity. Among the OTU members we studied, TRABID and virally encoded vOTU were exceptions. Toward our model substrates, vOTU demonstrated robust esterase and isopeptidase activity whereas TRABID only conferred esterase activity. However, as TRABID has been shown to mediate efficient isopeptidase activity toward Ub polymers, it would appear that it has the potential to confer dual chemical substrate specificity within cells. Most insights toward DUB substrates have been in the context of polyubiquitin linkage preference. In comparison, knowledge of physiological protein substrates is limited, and our findings imply that non-lysine ubiquitination sites must be considered when studying the majority of DUBs (USP, UCH, and MJD classes). We also establish that, in principle, Ub esters can be relatively long-lived inside cells, implying that ubiquitination of hydroxy amino acids can serve as a sustained cellular signal.

The discovery that MJD DUBs, and JOSD1 in particular, have selective Ub esterase activity was striking, suggestive of these DUBs being dedicated to specifically editing non-lysine ubiquitinated substrates. This is particularly evident from our observation that JOSD1 maintains specific esterase DUB activity in the context of the protein substrate UBE2J2. Furthermore, the molecular basis for UBE2J2 esterification activity, and whether any of the other ∼30 E2s might confer esterification activity, remain poorly understood. The modular nature of the RING E3 catalytic mechanism presents the possibility that, in principal, any of the ∼600 RING E3s could direct esterification of hydroxy amino acids within substrates. This is in further support of the prevalence of non-lysine ubiquitination being underestimated.

We identified features, within the MJD active site, that are crucial for their activity. The presence of hydrophobic residues adjacent to the catalytic cysteine appears to serve an important role in mediating selective esterification and esterase activity. The precise role these residues play might be further validated by structural studies. If such hydrophobic residues present within the active site are a universal feature of Ub cascade enzymes (E2s, E3s) that might also have non-lysine activity, then, where structural data exists, this feature could potentially be used to predict the existence of such enzymes.

Within the MJD family, ATXN3 represents the most well-studied enzyme as it is related to the development of spinocerebellar ataxia type-3, and its established esterase activity might be of patho-physiological relevance. Within the MJD family, JOSD1 and JOSD2 represent the least studied members. Despite their sequence homology, JOSD1 and JOSD2 seem to have distinct physiological functions and different catalytic efficiencies. JOSD2 has been found able to cleave ubiquitin chains in vitro while JOSD1 has been reported as having low activity or being inactive (44). These results are consistent with our finding that JOSD2 shows some degree of activity against the isopeptide-linked model-substrate. However, it should be noted that MJD DUB activity toward isopeptide-linked substrates has not been quantified.

The association of JOSD1 with the development of chemoresistance in gynecological cancer makes it the subject of a potential biomarker and therapeutic target. However, developing a robust assay for screening JOSD1 inhibitors would certainly be a challenge due to the low signal window obtained with conventional (iso)peptide-linked substrates. The substrates produced in our study, notably Ub-Thr-TAMRA, should facilitate the development of valuable assay platforms for inhibitor screening of MJD DUBs, an emerging class of therapeutic targets. The presence of UIM domains in ATXN3 and ATXN3L may have a significant effect on their cellular function. These could engage polyUb chains, thereby influencing cellular localization, and could also enhance catalytic efficiency through avidity effects. Furthermore, despite the striking specificity and high catalytic efficiency of JOSD1 threonine esterase activity, our data imply that catalytic efficiency toward serine substrates could be greater still and require quantification. Finally, the esterase activity conferred by MJD DUBs might allow it to be used in combination with DUBs with selective isopeptidase activity (e.g., OTUD3, OTUB2, and OTUD6) for diagnosis of Ub linkage chemistry within cellular substrates.

## Materials and Methods

Ubiquitin monomer, bovine serum albumin (BSA), Tris, dimethyl sulfoxide (DMSO), and DTT were purchased from Sigma-Aldrich. Ub-Lys-TAMRA-Gly was purchased from Boston Biochem (Boston, MA) (no. U-558). MALDI-TOF MS 1536 AnchorChip was purchased from Bruker Daltonics (Bremen, Germany).

The 2′,6′-dihydroxyacetophenone (DHAP) matrix was purchased from Tokyo Chemical Industry (product no. D1716). JOSD1 monoclonal antibody was purchased from Thermo Fisher (MA5-25365).

### Synthesis of Ub-Lys, Ub-Thr, and Ub-Glutathione Model Substrates.

Ub-Lys was prepared using a modification of GOPAL technology, which was developed for the production of defined isopeptide-linked Ub chains (18). In brief, an excess of *Nα*-acetyl-l-lysine was dissolved in DMSO together with a chemically protected Ub thioester protein in the presence of AgNO_3_ and *N*-hydroxysuccimimide as catalyst. After incubation at 23 °C, protein was precipitated with ice-cold diethylether and air-dried. Chemical protecting groups were removed as previously described (18), and deprotected protein was isolated by ether precipitation. Dried protein was dissolved in denaturing guanidinium chloride buffer and purified by RP-HPLC (17). Fractions containing Ub-Lysine were determined by liquid chromatography-mass spectrometry (LC-MS), freeze dried, and stored at −20 °C. Prior to DUB analysis, freeze-dried Ub-Lysine was dissolved in the minimum volume of DMSO. Protein folding was then initiated by the addition of 9 volume equivalents of PBS (53). This procedure was used to refold all model Ub substrates, and final concentration ranged from 0.8 to 3.0 mg/mL. For long-term storage, samples were aliquotted, frozen in liquid N_2_, and kept at −80 °C. Minor hydrolysis (∼10%) of ester-linked substrates was observed after several months storage.

Ub-Thr and Ub-Ser were prepared chemoenzymatically in reaction buffer consisting of Na_2_PO_4_ (40 mM), NaCl (150 mM), MgCl_2_ (5 mM), *Nα*-acetyl-l-threonine (50 mM), E1 Uba1 (500 nM), E2 UBE2D3 (10 μM), GST-MYCBP2cat (5 μM), Ub (50 μM), ATP (5 mM), and TCEP (0.5 mM) in a volume of 2.33 mL. The reaction was incubated at 37 °C for 1 to 2 h, and Ub-Thr was purified by RP-HPLC using a 20 to 50% gradient over 60 min (buffer A was 0.1% trifluoroacetic acid [TFA] in H_2_O, and buffer B was 0.1% TFA in acetonitrile). Substrates were reconstituted as described for Ub-Lys, and ester linkages were found to be stable in PBS at −80 °C for at least 6 mo.

To prepare Ub-Glutathione, Ub carrying a mercaptoethanesulfonic acid group was initially generated chemoenzymatically. Then, 1 mL of the chemoenzymatic reaction (20 mM Na_2_HPO_4_, pH 8, 10 mM MgCl_2_, 10 mM ATP, 467 μM Ub, 100 mM sodium mercaptoethanesulfonate, 250 nM E1) was incubated for 4 h at 37 °C. Ub-MESNA thioester was quantitatively produced and purified by semipreparative HPLC on a Dionex Ultimate 3000 System using a 250 mm × 21.2 mm Thermo Biobasic C4 column. A gradient of 10% mobile phase A to 80% mobile phase B over 30 min was applied (mobile phase A = 0.1% TFA in H_2_O, and buffer B = 0.1% TFA in acetonitrile). Fractions containing Ub-MESNA were then freeze-dried, yielding 3.7 mg. Ub-MESNA. (3.7 mg). was then dissolved in denaturing buffer containing reduced glutathione (200 mM Na_2_HPO_4_, pH 7.5, 6 M guanidinium chloride, 100 mM glutathione). The reaction was agitated for 2 h at 23 °C, allowing transthioesterification to proceed. Ub-Glutathione was then purified by semipreparative HPLC using a gradient of 20% buffer A to 70% buffer B over 60 min. Fractions containing Ub-Glutathione were determined by LC-MS, and product was freeze-dried (yield ∼1 mg) and stored at −20 °C.

### Synthesis of Ubiquitinated Peptide Model Substrates.

Ac-EGKGN-NH_2_, Ac-EGTGN-NH_2_, and Ac-EGSGN-NH_2_ were purchased from Bio-synthesis and resuspended in water to 250 mM final, the pH adjusted to 7 to 8 using 0.4 M NaOH. Ub-EGKGN and Ub-EGTGN/Ub-EGSGN were prepared chemoenzymatically using RNF4 and MYCBP2, respectively. RNF4 (10 μM) or GST-MYCBP2cat (5) (10 μM) were diluted in reaction buffer consisting of Na_2_PO_4_ (40 mM), NaCl (150 mM), MgCl_2_ (5 mM), *Nα*-acetyl-l-threonine (50 mM), E1 Uba1 (500 nM), E2 UBE2D3 (10 μM), Ub (50 μM), ATP (5 mM), and TCEP (0.5 mM). Reaction was incubated at 37 °C for 1 to 2 h, and Ub-EGKGN and Ub-EGTGN were purified by RP-HPLC using a 20 to 50% gradient over 60 min (buffer A was 0.1% TFA in H_2_O, and buffer B was 0.1% TFA in acetonitrile).

### Synthesis of Ub-Lys-TAMRA and Ub-Thr-TAMRA Fluorescent Substrates.

Initially, threonine and lysine were functionalized at the *Nα* position with TAMRA. A 10-fold molar excess of amino acid [l-threonine or *Nε-*(*t*-butyloxycarbonyl-)l-lysine] and *N*,*N*-diisopropylethylamine (DIEA) was mixed with 5/6 TAMRA-OSu (C1171; Thermo Fisher) in DMSO. After agitation for 24 h, reactions were diluted to 10% DMSO with H_2_O, and products were purified by RP-HPLC and lyophilized, yielding threonine-TAMRA and *Nε-*(*t*-butyloxycarbonyl)-Lys-TAMRA. To remove the *t*-butyloxycarbonyl protecting group from the lysine product, material was dissolved in the minimum volume of dichloromethane, which was subsequently diluted to 60% with TFA. Solvent was removed with a stream of air and repurified by RP-HPLC, yielding Lys-TAMRA. Fluorescent Ub-Lys-TAMRA and Ub-Thr-TAMRA substrates were prepared as described for Ub-Lys and Ub-Thr, but *Nα*-acetyl-l-lysine and l-threonine were substituted for Lys-TAMRA and Thr-TAMRA, respectively.

### FP Assay.

Ub-K/T-TAMRA (final concentration 0.250 to 40 μM for kinetic calculation, 0.250 μM for standard assay) were diluted in FP buffer (50 mM Tris⋅HCl, pH 7.5, 150 mM NaCl, 1 mM DTT, 0.01% BSA) aliquotted into a 384-well plate (black low volume, round bottom; Corning). DUBs were diluted at the indicated final concentration in FP buffer and added into each well. FP decay was measured with a cycle time of 30 s for 60 cycles at 30 °C using a plate reader (Pherarastar; BMG Labtech). Normalized parallel and perpendicular fluorescence intensities were used for further calculations. Graphpad Prism 8.0 was used to fit the data into Michaelis–Menten equations and calculate kinetic parameters. Observed first order rate constants (*k*_*obs*_) were approximated using the half-life equation *t*_*1/2*_ = ln2/*k*_obs_.

### Gel-Based Ubiquitinated Peptide Cleavage Assay.

DUBs (750 nM final concentration) and Ub-EGKGN/Ub-EGTGN (5 μM final concentration) were diluted in 50 mM Tris⋅HCl, pH 7.0, 50 mM NaCl_2_, and 1 mM DTT. Reaction was incubated for the indicated time points at 30° and stopped by adding 1× final LDS-NuPAGE sample buffer. Samples were run on 1-mm, 4 to 12% Bis-Tris Protein Gels for 45 min at 200 V and blue Coomassie stained. Silver staining was also carried out in accordance with manufacturer protocols (Thermo Scientific Pierce Silver Stain Kit).

### Target Spotting and MALDI Mass Spectrometry Analysis.

The DHAP matrix solution was prepared as previously described (17). Briefly, 7.6 mg of DHAP were resuspended in 375 μL of LC-MS grade ethanol and 125 μL of an aqueous solution of 25 mg/mL diammoniumhydrogen citrate (25102-500G-R; Sigma Aldrich). MALDI-target spotting and MS analysis were performed as previously described (54). Briefly, a Mosquito nanoliter dispenser (TTP Labtech, Hertfordshire, UK) was employed to mix 1.2 μL of each reaction with 1.2 μL of DHAP matrix solution. Then, 200 nL of matrix/assay mixture from each sample were spotted onto a 1536 AnchorChip MALDI target. Spotted MALDI-targets were air dried prior to MALDI-TOF MS analysis. All samples were acquired as previously reported on a Rapiflex MALDI-TOF mass spectrometer (Bruker Daltonics, Bremen, Germany) equipped with Compass for FlexSeries 2.0, FlexControl, and FlexAnalysis software (version 4.0). Peak intensities were exported as. csv file using FlexAnalysis. An in-house Windows batch script was used to report peak intensities into an Excel grid with the same geometry as for the MALDI-target. Because of the intrinsic variability of MALDI-TOF ionization, for diUb substrates, the mono Ub product was quantified by reference to an internal standard consisting of ^15^N-labeled Ub, allowing determination of percent cleavage (17). For Ub-W or Ub-Glutathione substrates, the mono Ub product was also quantified by reference to the isotopic standard, but, to account for the single molecule of Ub generated upon cleavage of a substrate molecule, the following equation was applied:

% Ub Substrate Cleavage= ((Area Ub)/(AreaN15 Ub)×[N15 Ub])/[Ub Substrate]×100

As the mass of the Ub-Thr substrate overlapped with the ^15^N-labeled Ub, standard curves were generated for Ub-Lys and Ub-Thr using defined product/substrate ratios allowing determination of percent of cleavage. In the case of Ub-Ser, the standard curve generated for Ub-Thr was applied.

### MALDI-TOF DUB Assay.

Enzymes and substrates were freshly prepared in the reaction buffer (40 mM Tris⋅HCl, pH 7.6, 5 mM DTT, 0.005% BSA). DUBs were diluted at the indicated concentrations (*SI Appendix*, Table S1), and 5.8 μL of reaction buffer and 3 μL of diluted DUBs were aliquotted in a Greiner 384-well plate, flat round bottom, low binding. Then, 1.2 μL of either Ub dimers, Ub-W, Ub-Lys, Ub-Thr, Ub-Ser, or Ub-Glutathione was added to the reaction mixture at final concentration of 1.4, 2.75, 2.75, 2.75, 2.75, and 2.75 µM, respectively. DUBs tested for their ability to cleave Ub-Glutathione were previously dialyzed against 1× PBS buffer supplemented with 0.5 mM TCEP. DTT was also withheld from the DUB assay buffer. The reaction was incubated at 30 °C and stopped by adding 2.5 μL of 10% TFA at the indicated time points. ^15^N-labeled Ub was added as internal standard only to the reaction controls using Ub dimers, Ub-W, or Ub-Glutathione as substrate.

### Cellular Assessment of Ubiquitin Ester Stability.

E2_Ser_∼Ub was prepared using a Cys85Ser mutant of an N-terminal His-tagged clone of the E2 enzyme UbcH5c. Ester loading was carried out enzymatically, and product was purified as previously described, with the exception that the reaction was performed at pH 7.5 (39). Low passage HEK293 cells (10^6^) were resuspended in 30 μL of Neon Electroporation Buffer R containing 26 µg (3 μL of 8.6 mg/mL stock) E2_Ser_∼Ub. The mixture (20 μL) was directly transferred into prewarmed Dulbecco’s modified Eagle’s medium (DMEM) supplemented with 10% (vol/vol) fetal bovine serum (FBS), 2.0 mM ʟ-glutamine, and lacking antibiotics, and incubated at 37 °C/5% CO_2,_ while another 20 μL was electroporated using the Neon Transfection System (2 × 1,400 V, 20 ms) in 10-µL tips, before transfer into prewarmed media and incubation at 37 °C/5% CO_2_. The experiment was replicated in parallel, using HEK293 pretreated with MG132 (20 μM) for 30 min, and cells were subsequently incubated in media containing 20 μM MG132. Then, 600,000 cells were collected at intervals postelectroporation, washed thrice in ice-cold PBS, and lysed in ice-cold lysis buffer (50 mM Tris⋅HCl, pH 7.5, 10 mM sodium 2-glycerophosphate, 50 mM sodium fluoride, 5.0 mM sodium pyrophosphate, 1.0 mM sodium orthovanadate, 0.27 M sucrose, 50 mM NaCl, 0.2 mM phenylmethanesulfonyl fluoride [PMSF], 1.0 mM benzamidine, 10 mM TCEP, 1% Nonidet P-40) on ice for 30 min. Lysates were clarified by centrifugation at 4 °C for 20 min at 21,100 × *g*. Supernatants were collected, and protein concentration was determined by Bradford assay. Immunoblotting was carried out, and membranes were probed with antibodies against the His tag on the E2 protein in E2_Ser_∼Ub (631212; Clontech) and tubulin (66031-1-Ig; Proteintech) as a loading control.

### UBE2J2-Based DUB Activity Assay.

A UBE2J2 automodification reaction was performed by incubating E1 (200 nM), Ub (50 µM), and recombinant His-UBE2J2 (10 µM) (5) in reaction Buffer (1× PBS, 20 mM MgCl_2_, 2 mM ATP, 1 mM TCEP) for 30 min at 30 °C in a final volume of 100 µL. Compound 1 E1 inhibitor was added at a final concentration of 25 µM, and the reaction was incubated for 15 min at 30 °C. The reaction was then subaliquotted and treated with either JOSD1 (3 µM), USP2 (3 µM), or 1× PBS buffer for 30 min at 30 °C. Reactions were stopped by addition of 4× lithium dodecyl sulfate loading buffer (Thermo Fisher Scientific) plus BME and supplemented with either water or hydroxylamine (0.5 M). Samples where incubated for 30 min at 37 °C and resolved on a 4 to 12% Bis-Tris gel at 125 V for 1.4 h. Protein bands were either visualized by Coomassie stain or by standard Western blot procedure (anti-ubiquitin antibody, cat. no. 646302, monoclonal, mouse, 0.1 µg/mL; BioLegend).

### Transient Overexpression of JOSD1 in HEK293 Cells.

HEK293 cells (10^6^) were seeded in a 10-cm dish and incubated for 16 h at 37 °C, 5% CO_2_. At ∼70% confluency, JOSD1-HA plasmid (7 μg; vector pCMV-HA-C) was transfected using Lipofectamine 2000, and cells were incubated for 24 h. Proteasome inhibitor MG-132 (10 μM, 474790; Millipore-Sigma) was added to cells 5 h before lysis. Cells were lysed in buffer (50 mM Tris⋅HCl, 250 mM NaCl2, 0.1% Nonidet P-40) supplemented with cOmplete, Mini, (ethylenedinitrilo)tetraacetic acid (EDTA)-free Protease Inhibitor Tablets (11836170001; Roche), benzamidine (1 mM), PMSF (0.25 mM), and MG132 (10 μM). Lysates were clarified by centrifugation and quantified by Bradford assay. JOSD1 detection by immunoblotting was performed using JOSD1 monoclonal antibody (OTI3B11; Thermo Fisher Scientific) and standard immunoblotting procedures.

## Supplementary Material

Supplementary File

## Data Availability

All study data are included in the article and *SI Appendix*.

## Change History

March 2, 2021: The license for this article has been updated.

## References

[1] E. Oh, D. Akopian, M. Rape, Principles of ubiquitin-dependent signaling. Annu. Rev. Cell Dev. Biol. 34, 137–162 (2018). 30110556 10.1146/annurev-cellbio-100617-062802

[2] A. Hershko, A. Ciechanover, The ubiquitin system. Annu. Rev. Biochem. 67, 425–479 (1998). 9759494 10.1146/annurev.biochem.67.1.425

[3] R. J. Deshaies, C. A. Joazeiro, RING domain E3 ubiquitin ligases. Annu. Rev. Biochem. 78, 399–434 (2009). 19489725 10.1146/annurev.biochem.78.101807.093809

[4] N. Zheng, N. Shabek, Ubiquitin ligases: Structure, function, and regulation. Annu. Rev. Biochem. 86, 129–157 (2017). 28375744 10.1146/annurev-biochem-060815-014922

[5] K. C. Pao., Activity-based E3 ligase profiling uncovers an E3 ligase with esterification activity. Nature 556, 381–385 (2018). 29643511 10.1038/s41586-018-0026-1

[6] Y. T. Kwon, A. Ciechanover, The ubiquitin code in the ubiquitin-proteasome system and autophagy. Trends Biochem. Sci. 42, 873–886 (2017). 28947091 10.1016/j.tibs.2017.09.002

[7] M. J. Clague, S. Urbé, D. Komander, Breaking the chains: Deubiquitylating enzyme specificity begets function. Nat. Rev. Mol. Cell Biol. 20, 338–352 (2019). 30733604 10.1038/s41580-019-0099-1

[8] E. Goto., c-MIR, a human E3 ubiquitin ligase, is a functional homolog of herpesvirus proteins MIR1 and MIR2 and has similar activity. J. Biol. Chem. 278, 14657–14668 (2003). 12582153 10.1074/jbc.M211285200

[9] K. Cadwell, L. Coscoy, Ubiquitination on nonlysine residues by a viral E3 ubiquitin ligase. Science 309, 127–130 (2005). 15994556 10.1126/science.1110340

[10] X. Wang., Ubiquitination of serine, threonine, or lysine residues on the cytoplasmic tail can induce ERAD of MHC-I by viral E3 ligase mK3. J. Cell Biol. 177, 613–624 (2007). 17502423 10.1083/jcb.200611063 PMC2064207

[11] L. Jin, A. Williamson, S. Banerjee, I. Philipp, M. Rape, Mechanism of ubiquitin-chain formation by the human anaphase-promoting complex. Cell 133, 653–665 (2008). 18485873 10.1016/j.cell.2008.04.012 PMC2696189

[12] X. Wang., Ube2j2 ubiquitinates hydroxylated amino acids on ER-associated degradation substrates. J. Cell Biol. 187, 655–668 (2009). 19951915 10.1083/jcb.200908036 PMC2806592

[13] B. Grill, R. K. Murphey, M. A. Borgen, The PHR proteins: Intracellular signaling hubs in neuronal development and axon degeneration. Neural Dev. 11, 8 (2016). 27008623 10.1186/s13064-016-0063-0 PMC4806438

[14] F. Tokunaga., Involvement of linear polyubiquitylation of NEMO in NF-kappaB activation. Nat. Cell Biol. 11, 123–132 (2009). 19136968 10.1038/ncb1821

[15] I. R. Kelsall, J. Zhang, A. Knebel, J. S. C. Arthur, P. Cohen, The E3 ligase HOIL-1 catalyses ester bond formation between ubiquitin and components of the Myddosome in mammalian cells. Proc. Natl. Acad. Sci. U.S.A. 116, 13293–13298 (2019). 31209050 10.1073/pnas.1905873116 PMC6613137

[16] H. Sun, R. Meledin, S. M. Mali, A. Brik, Total chemical synthesis of ester-linked ubiquitinated proteins unravels their behavior with deubiquitinases. Chem. Sci. (Camb.) 9, 1661–1665 (2018). 10.1039/c7sc04518b PMC5887810 29675213

[17] M. S. Ritorto., Screening of DUB activity and specificity by MALDI-TOF mass spectrometry. Nat. Commun. 5, 4763 (2014). 25159004 10.1038/ncomms5763 PMC4147353

[18] S. Virdee, Y. Ye, D. P. Nguyen, D. Komander, J. W. Chin, Engineered diubiquitin synthesis reveals Lys29-isopeptide specificity of an OTU deubiquitinase. Nat. Chem. Biol. 6, 750–757 (2010). 20802491 10.1038/nchembio.426

[19] J. P. Tam, Q. Yu, Y. A. Lu, Tandem peptide ligation for synthetic and natural biologicals. Biologicals 29, 189–196 (2001). 11851315 10.1006/biol.2001.0292

[20] T. Hadari, J. V. Warms, I. A. Rose, A. Hershko, A ubiquitin C-terminal isopeptidase that acts on polyubiquitin chains. Role in protein degradation. J. Biol. Chem. 267, 719–727 (1992). 1309773

[21] M. Akutsu, Y. Ye, S. Virdee, J. W. Chin, D. Komander, Molecular basis for ubiquitin and ISG15 cross-reactivity in viral ovarian tumor domains. Proc. Natl. Acad. Sci. U.S.A. 108, 2228–2233 (2011). 21266548 10.1073/pnas.1015287108 PMC3038727

[22] H. Tran, F. Hamada, T. Schwarz-Romond, M. Bienz, Trabid, a new positive regulator of Wnt-induced transcription with preference for binding and cleaving K63-linked ubiquitin chains. Genes Dev. 22, 528–542 (2008). 18281465 10.1101/gad.463208 PMC2238673

[23] J. Jin., Epigenetic regulation of the expression of Il12 and Il23 and autoimmune inflammation by the deubiquitinase Trabid. Nat. Immunol. 17, 259–268 (2016). 26808229 10.1038/ni.3347 PMC4755875

[24] J. D. Licchesi., An ankyrin-repeat ubiquitin-binding domain determines TRABID’s specificity for atypical ubiquitin chains. Nat. Struct. Mol. Biol. 19, 62–71 (2011). 22157957 10.1038/nsmb.2169 PMC5260945

[25] Y. Zhu., Trabid inhibits hepatocellular carcinoma growth and metastasis by cleaving RNF8-induced K63 ubiquitination of Twist1. Cell Death Differ. 26, 306–320 (2019). 29748601 10.1038/s41418-018-0119-2 PMC6329825

[26] T. E. T. Mevissen, D. Komander, Mechanisms of deubiquitinase specificity and regulation. Annu. Rev. Biochem. 86, 159–192 (2017). 28498721 10.1146/annurev-biochem-061516-044916

[27] Y. A. Kristariyanto, S. A. Abdul Rehman, S. Weidlich, A. Knebel, Y. Kulathu, A single MIU motif of MINDY-1 recognizes K48-linked polyubiquitin chains. EMBO Rep. 18, 392–402 (2017). 28082312 10.15252/embr.201643205 PMC5331195

[28] D. Kwasna., Discovery and characterization of ZUFSP/ZUP1, a distinct deubiquitinase class important for genome stability. Mol. Cell 70, 150–164.e6 (2018). 29576527 10.1016/j.molcel.2018.02.023 PMC5896202

[29] T. Hermanns., A family of unconventional deubiquitinases with modular chain specificity determinants. Nat. Commun. 9, 799 (2018). 29476094 10.1038/s41467-018-03148-5 PMC5824887

[30] P. Haahr., ZUFSP deubiquitylates K63-linked polyubiquitin chains to promote genome stability. Mol. Cell 70, 165–174.e6 (2018). 29576528 10.1016/j.molcel.2018.02.024

[31] D. S. Hewings., Reactive-site-centric chemoproteomics identifies a distinct class of deubiquitinase enzymes. Nat. Commun. 9, 1162 (2018). 29563501 10.1038/s41467-018-03511-6 PMC5862848

[32] Y. Sato., Structural basis for specific cleavage of Lys 63-linked polyubiquitin chains. Nature 455, 358–362 (2008). 18758443 10.1038/nature07254

[33] D. Wang., Human carboxylesterases: A comprehensive review. Acta Pharm. Sin. B 8, 699–712 (2018). 30245959 10.1016/j.apsb.2018.05.005 PMC6146386

[34] D. Clift. A method for the acute and rapid degradation of endogenous proteins. Cell 171, 1692–1706.e18 (2017). 29153837 10.1016/j.cell.2017.10.033 PMC5733393

[35] M. J. Eddins, C. M. Carlile, K. M. Gomez, C. M. Pickart, C. Wolberger, Mms2-Ubc13 covalently bound to ubiquitin reveals the structural basis of linkage-specific polyubiquitin chain formation. Nat. Struct. Mol. Biol. 13, 915–920 (2006). 16980971 10.1038/nsmb1148

[36] P. P. Geurink, F. El Oualid, A. Jonker, D. S. Hameed, H. Ovaa, A general chemical ligation approach towards isopeptide-linked ubiquitin and ubiquitin-like assay reagents. ChemBioChem 13, 293–297 (2012). 22213387 10.1002/cbic.201100706 PMC3488293

[37] Z. S. Zhou., Competitive oxidation and ubiquitylation on the evolutionarily conserved cysteine confer tissue-specific stabilization of Insig-2. Nat. Commun. 11, 379 (2020). 31953408 10.1038/s41467-019-14231-w PMC6969111

[38] O. N. Burchak., Chemoenzymatic ubiquitination of artificial substrates. ChemBioChem 7, 1667–1669 (2006). 17009276 10.1002/cbic.200600283

[39] A. Plechanovová., Mechanism of ubiquitylation by dimeric RING ligase RNF4. Nat. Struct. Mol. Biol. 18, 1052–1059 (2011). 21857666 10.1038/nsmb.2108 PMC3326525

[40] S. M. Nijman., A genomic and functional inventory of deubiquitinating enzymes. Cell 123, 773–786 (2005). 16325574 10.1016/j.cell.2005.11.007

[41] B. Burnett, F. Li, R. N. Pittman, The polyglutamine neurodegenerative protein ataxin-3 binds polyubiquitylated proteins and has ubiquitin protease activity. Hum. Mol. Genet. 12, 3195–3205 (2003). 14559776 10.1093/hmg/ddg344

[42] C. A. Matos, S. de Macedo-Ribeiro, A. L. Carvalho, Polyglutamine diseases: The special case of ataxin-3 and Machado-Joseph disease. Prog. Neurobiol. 95, 26–48 (2011). 21740957 10.1016/j.pneurobio.2011.06.007

[43] L. Masino., Domain architecture of the polyglutamine protein ataxin-3: A globular domain followed by a flexible tail. FEBS Lett. 549, 21–25 (2003). 12914917 10.1016/s0014-5793(03)00748-8

[44] T. Seki., JosD1, a membrane-targeted deubiquitinating enzyme, is activated by ubiquitination and regulates membrane dynamics, cell motility, and endocytosis. J. Biol. Chem. 288, 17145–17155 (2013). 23625928 10.1074/jbc.M113.463406 PMC3682520

[45] X. Wu., JOSD1 inhibits mitochondrial apoptotic signalling to drive acquired chemoresistance in gynaecological cancer by stabilizing MCL1. Cell Death Differ. 27, 55–70 (2019). 31043700 10.1038/s41418-019-0339-0 PMC7206032

[46] X. Wang., JOSD1 negatively regulates type-I interferon antiviral activity by deubiquitinating and stabilizing SOCS1. Viral Immunol. 30, 342–349 (2017). 28355105 10.1089/vim.2017.0015

[47] S. V. Todi., Ubiquitination directly enhances activity of the deubiquitinating enzyme ataxin-3. EMBO J. 28, 372–382 (2009). 19153604 10.1038/emboj.2008.289 PMC2646149

[48] S. D. Weeks, K. C. Grasty, L. Hernandez-Cuebas, P. J. Loll, Crystal structure of a josephin-ubiquitin complex: Evolutionary restraints on ataxin-3 deubiquitinating activity. J. Biol. Chem. 286, 4555–4565 (2011). 21118805 10.1074/jbc.M110.177360 PMC3039388

[49] M. Renatus., Structural basis of ubiquitin recognition by the deubiquitinating protease USP2. Structure 14, 1293–1302 (2006). 16905103 10.1016/j.str.2006.06.012 PMC7126176

[50] C. H. Emmerich., Activation of the canonical IKK complex by K63/M1-linked hybrid ubiquitin chains. Proc. Natl. Acad. Sci. U.S.A. 110, 15247–15252 (2013). 23986494 10.1073/pnas.1314715110 PMC3780889

[51] P. P. Geurink., Development of diubiquitin-based FRET probes to quantify ubiquitin linkage specificity of deubiquitinating enzymes. ChemBioChem 17, 816–820 (2016). 26996281 10.1002/cbic.201600017 PMC4922411

[52] L. A. Kelley, S. Mezulis, C. M. Yates, M. N. Wass, M. J. Sternberg, The Phyre2 web portal for protein modeling, prediction and analysis. Nat. Protoc. 10, 845–858 (2015). 25950237 10.1038/nprot.2015.053 PMC5298202

[53] F. El Oualid., Chemical synthesis of ubiquitin, ubiquitin-based probes, and diubiquitin. Angew. Chem. Int. Ed. Engl. 49, 10149–10153 (2010). 21117055 10.1002/anie.201005995 PMC3021723

[54] V. De Cesare., The MALDI-TOF E2/E3 ligase assay as universal tool for drug discovery in the ubiquitin pathway. Cell Chem. Biol. 25, 1117–1127.e4 (2018). 30017913 10.1016/j.chembiol.2018.06.004 PMC6162346

[55] J. E. Brownell., Substrate-assisted inhibition of ubiquitin-like protein-activating enzymes: The NEDD8 E1 inhibitor MLN4924 forms a NEDD8-AMP mimetic in situ. Mol. Cell 37, 102–111 (2010). 20129059 10.1016/j.molcel.2009.12.024

