# Supplementary material for: Deubiquitinating enzyme amino acid profiling reveals a class of ubiquitin esterases
Source: Proc Natl Acad Sci U S A. 2021 Jan 21;118(4):e2006947118. doi: 10.1073/pnas.2006947118 (PMC7848729; doi:10.1073/pnas.2006947118)
Supplement: Supplementary File [file pnas.2006947118.sapp.pdf]

Deubiquitinating Enzyme Amino Acid Profiling Reveals a Class of Ubiquitin Esterases

SI Appendix

Virginia De Cesare, Daniel Carbajo Lopez, Peter D. Mabbitt, Adam J. Fletcher, Mathieu Soetens, Odetta Antico, Nicola T. Wood and Satpal Virdee*

MRC Protein Phosphorylation and Ubiquitylation Unit, University of Dundee, Scotland, UK, DD1 5EH.

* Corresponding author
Email: s.s.virdee@dundee.ac.uk
Tel: +44 (0)1382 388738
Fax: +44 (0)1382 388500

Supplementary Methods

Protein Expression and Purification

The expression and production of most of the DUBs, and ^{15}N Ub, were carried out as previously described[17]. The additional DUBs were expressed as follows. USP11; USP30 (57-517); USP45; UCH-L3, UCH-L5, MINDY2, MINDY3, MINDY4, ZUFSP, ATXN3 and ATXN3L (WT and mutants) and ZUFSP were cloned into pGEX-vectors, expressed in BL21(DE3) cells. Expression was induced with 0.05 - 0.25 mM isopropyl-β-D-thiogalactoside (IPTG) and allowed to proceed for 16h at 16 °C, followed by affinity purification against GSH-Agarose using standard protocols. Similarly, UCH-L1, USP47, JOSD1 (WT and mutants) and JOSD2, were cloned into pET-vectors, expressed in BL21(DE3) cells and affinity purified on nickel nitrilotriacetic acid (Ni-NTA) agarose. MINDY1 was cloned into pMex-vector, expressed in BL21(DE3) cells and affinity purified on amylose-agarose using standard protocols. USP12 and USP46 were co-expressed with WDR48 /UAF1 as 6His-tagged fusion proteins in Sf21 cells and purified over Ni-NTA agarose using standard protocols. The peptide-linked UCH substrate Ub-W was expressed in BL21(DE3). Briefly, expression was induced when cells were at an optical density of 1.0 at 600 nM with 1mM IPTG and left for 3.5h at 37 °C. The cells were harvested in MilliQ water, resuspended and frozen in liquid nitrogen. After thawing, the suspension was sonicated and clarified by centrifugation (38000 x g for 30 min at 4 °C). The lysate was titrated to pH 4.3 with 7% perchloric acid and left overnight. Precipitated proteins were removed by centrifugation and the clarified solution was subjected to cation exchange chromatography on a Source S HR10/10 column using a shallow NaCl gradient. The protein was concentrated to 50mg/ml and stabilised by supplementation with 50 mM Tris and titrated to final pH of 8.0 with HCl.

Tissue Harvesting and Lysates preparation

Brain tissues were rapidly excised and the sub-regions microdissection was performed with an ice cooling plate under stereomicroscopy. Upon isolation, brain subregions and peripheral tissues were collected in a single 1.5 ml microcentrifuge tube and snap-frozen in

liquid nitrogen. Tissue samples were stored at −80°C until ready for processing. All tissues were weighed and defrosted on wet ice in fivefold mass excess of freshly prepared, ice-cold lysis buffer containing: 50 mM Tris/HCl pH 7.5, 1 mM EDTA pH 8.0, 1 mM ethylene glycol-bis(β-aminoethyl ether)-*N,N,N′,N′*-tetraacetic acid (EGTA) pH 8.0, 1% Triton X-100, 0.25 M sucrose, 1 mM sodium orthovanadate, 50 mM NaF, 10 mM sodium glycerolphosphate, 10 mM sodium pyrophosphate, 200 mM 2-chloroacetamide, phosphatase inhibitor cocktail 3 (Sigma- Aldrich) and complete protease inhibitor cocktail (Roche). Tissue homogenization was performed using a probe sonicator at 4°C (Branson Instruments), with 10% amplitude and 2 cycles sonication (10 seconds on, 10 seconds off). Crude lysates were incubated at 4°C for 30 min on ice, before clarification by centrifugation at 20,800 x g in an Eppendorf 5417R centrifuge for 30 min at 4°C. Supernatants were collected and protein concentration was determined using a Bradford kit (Pierce).

Supplementary Figures

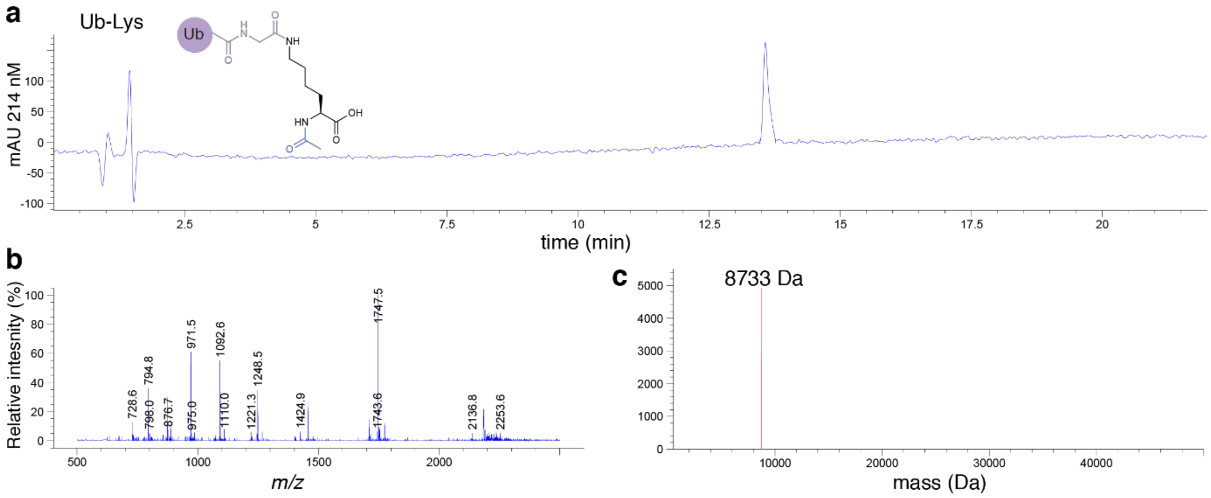

Figure S1. LC-MS characterization of purified Ub-Lys. (a) HPLC chromatogram for purified Ub-Lys monitoring at 214 nm. **(b)** Electrospray ionization mass spectrum for Ub-Lys. **(c)** Deconvoluted mass spectrum for Ub-Lys. Theoretical mass = 8735.06; observed mass = 8733 Da.

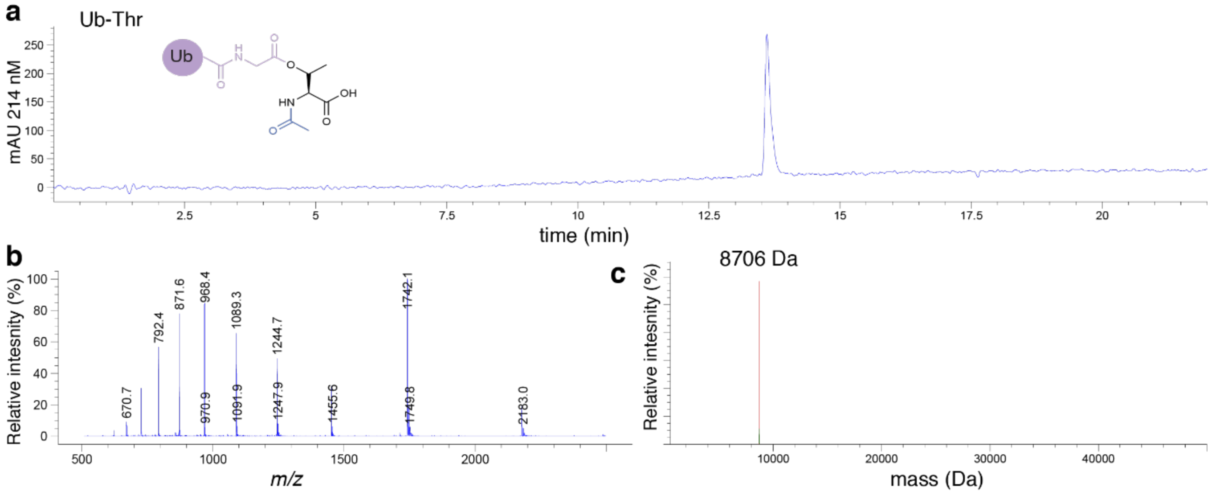

Figure S2. LC-MS characterization of purified Ub-Thr. (a) HPLC chromatogram for purified Ub-Thr monitoring at 214 nm. **(b)** Electrospray ionization mass spectrum for Ub-Thr. **(c)** Deconvoluted mass spectrum for Ub-Thr. Theoretical mass = 8708.00; observed mass = 8706 Da.

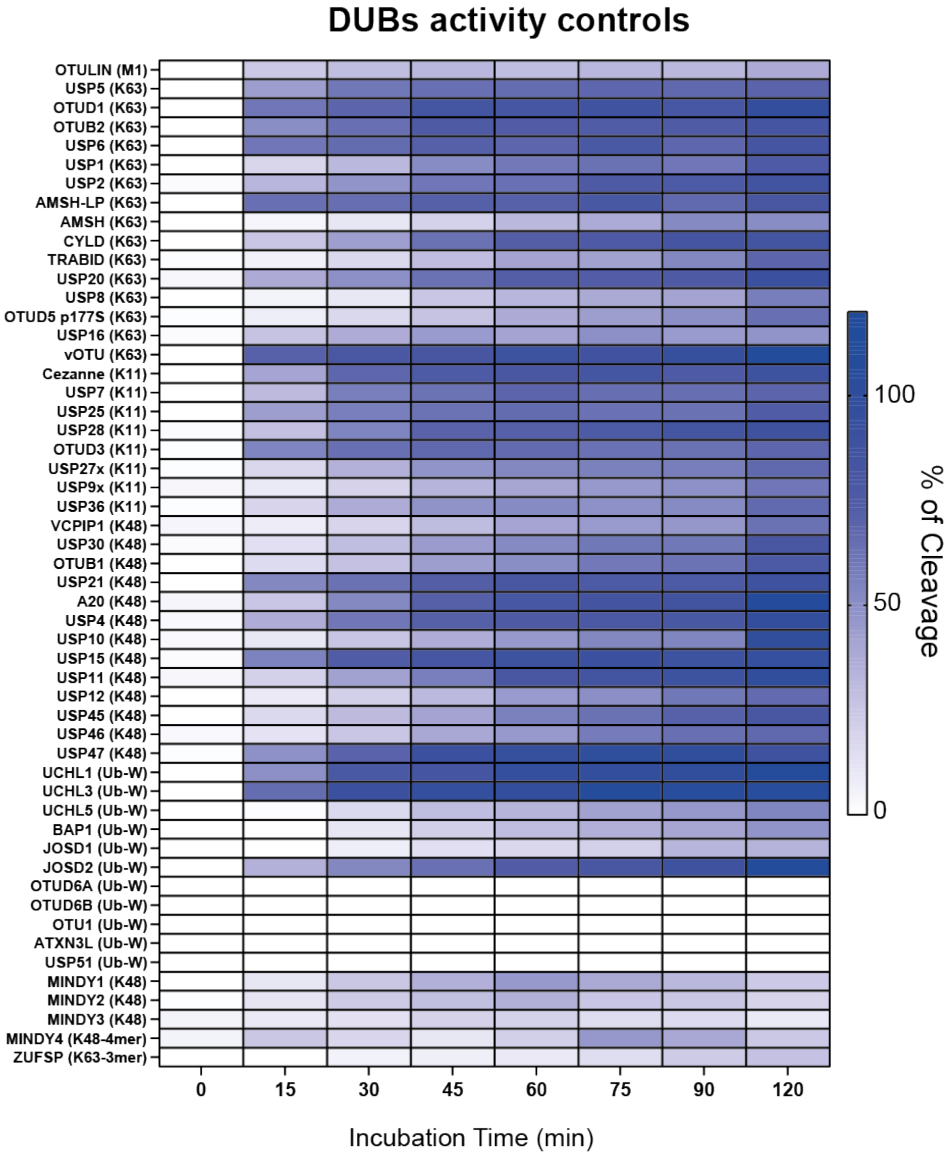

Figure S3. MALDI-TOF DUBs assay: enzymatic activity control: DUBs were tested in parallel for their activity with Ub dimer of linkage type known to be cleaved by the DUB under investigation (indicated as M1-K11-K48-K63). For DUBs that demonstrate negligible activity with Ub dimers, Ub with a C-terminal peptide-linked tryptophan was employed (Ub-W). The DUBs JOSD1, OTU1, OTUD6A and OTUD6B demonstrate no detectable activity towards any of the isopeptide-linked Ub dimers nor the peptide-linked substrate.

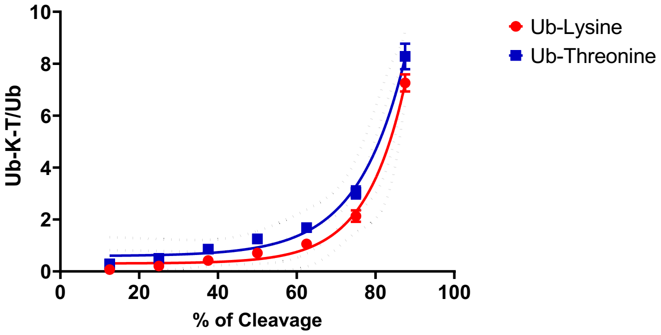

Figure S4. Standard curve for assessment of % substrate cleavage using MALDI-TOF DUB assay. Defined ratios of Ub-Lys (or Ub-Thr) substrate were mixed with free Ub that simulated intermediate states of substrate cleavage. Extrapolation of product/substrate ratios from DUB assays to the standard curve allowed assessment of % substrate cleaved.

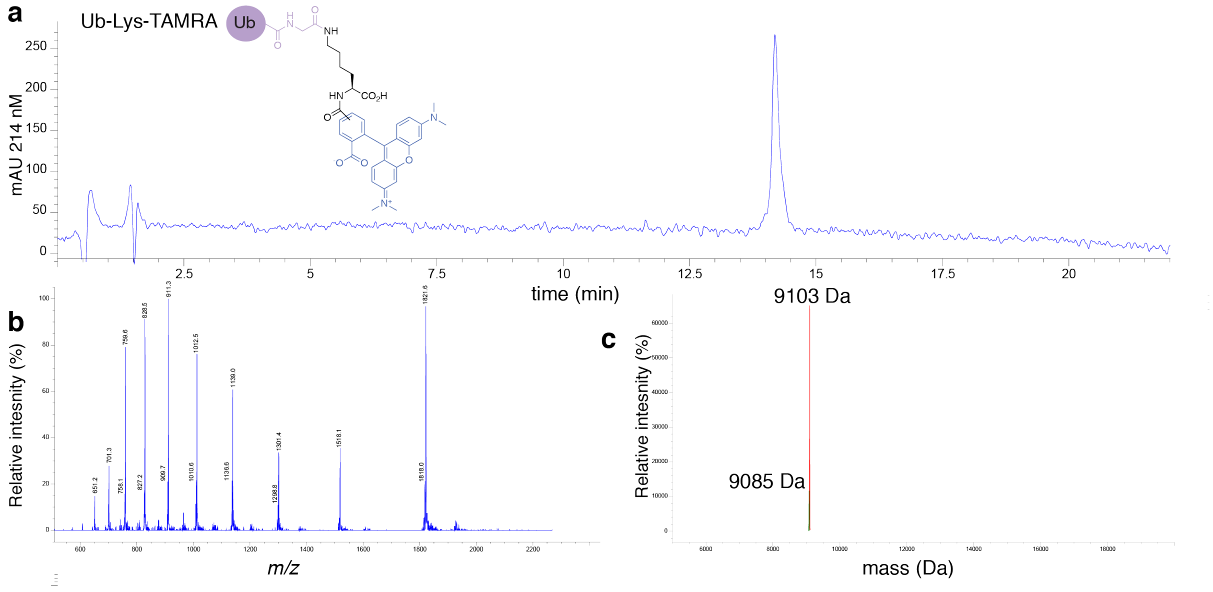

Figure S5. LC-MS characterization of purified Ub-Lys-TAMRA. (a) HPLC chromatogram for purified Ub-Lys-TAMRA monitoring at 214 nm. **(b)** Electrospray ionization mass spectrum for Ub-Lys-TAMRA. **(c)** Deconvoluted mass spectrum for Ub-Lys-TAMRA. Theoretical mass = 9105.46; observed mass = 9103 Da. Minor peak with mass of 9085 Da corresponds to a dehydration product.

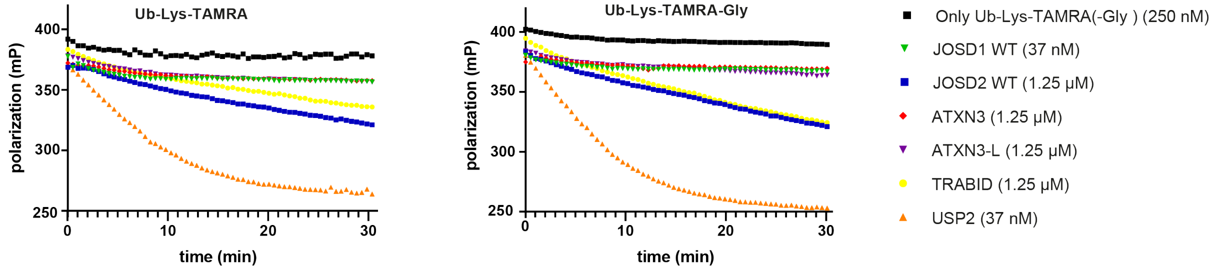

Figure S6. Comparison of activity of selected DUBs towards Ub-Lys-TAMRA and Ub-Lys-TAMRA-Gly.

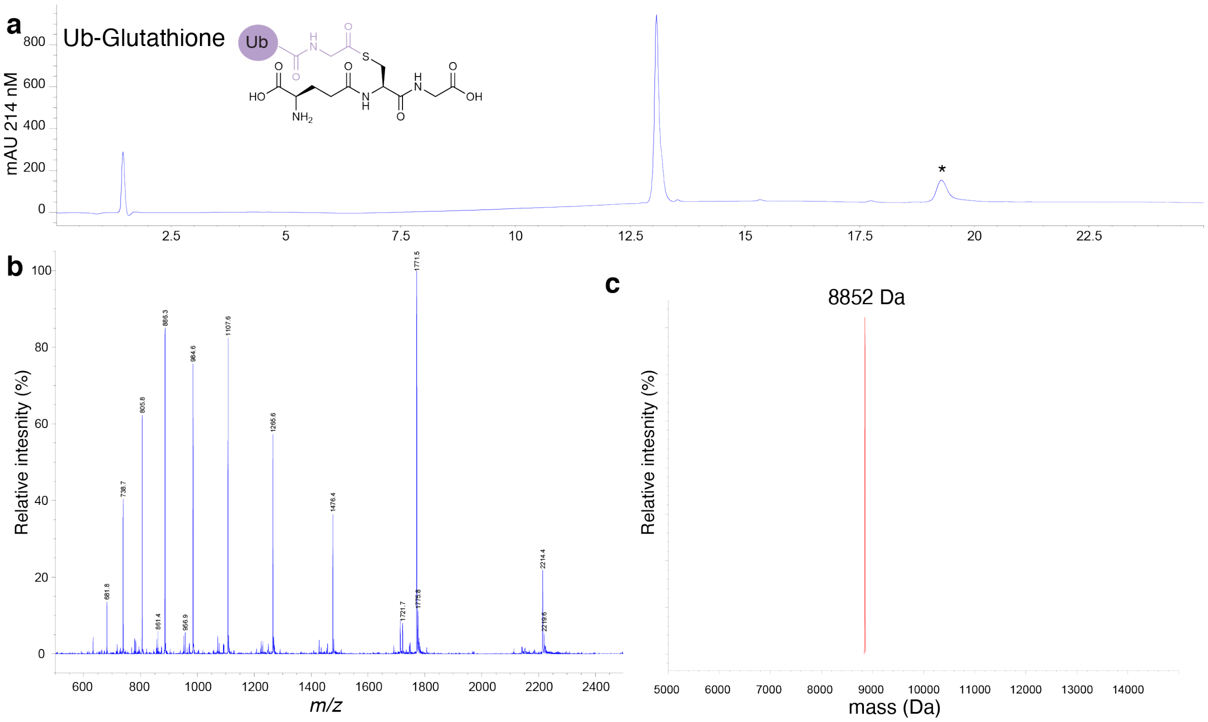

Figure S7. LC-MS characterization of purified Ub-Glutathione (a) HPLC chromatogram for purified Ub-Glutathione monitoring at 214 nm. Asterisk corresponds to a polymer contaminant of the LC-MS instrument rather than the sample. **(b)** Electrospray ionization mass spectrum for Ub-Glutathione. **(c)** Deconvoluted mass spectrum for Ub-Glutathione. Theoretical mass = 8854.15; observed mass = 8852 Da.

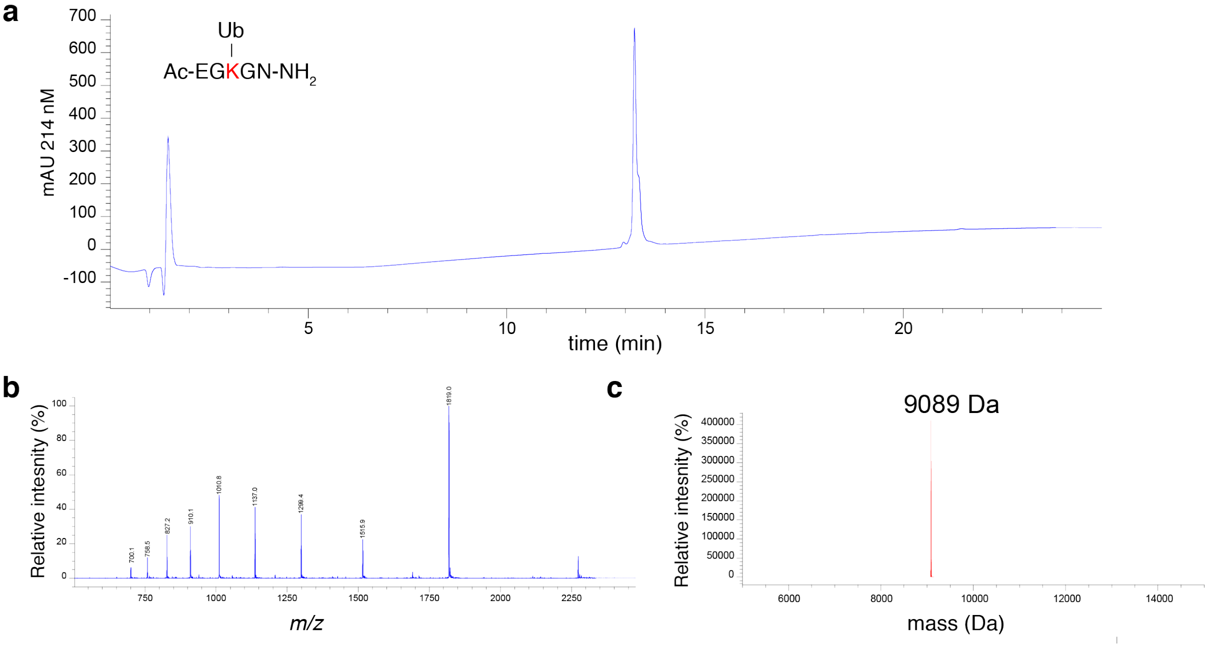

Figure S8. LC-MS characterization of purified Ub-EGKGN. (a) HPLC chromatogram for purified Ub-EGKGN monitoring at 214 nm. **(b)** Electrospray ionization mass spectrum for Ub-EGKGN. **(c)** Deconvoluted mass spectrum for Ub-EGKGN. Theoretical mass = 9091.52; observed mass = 9089 Da.

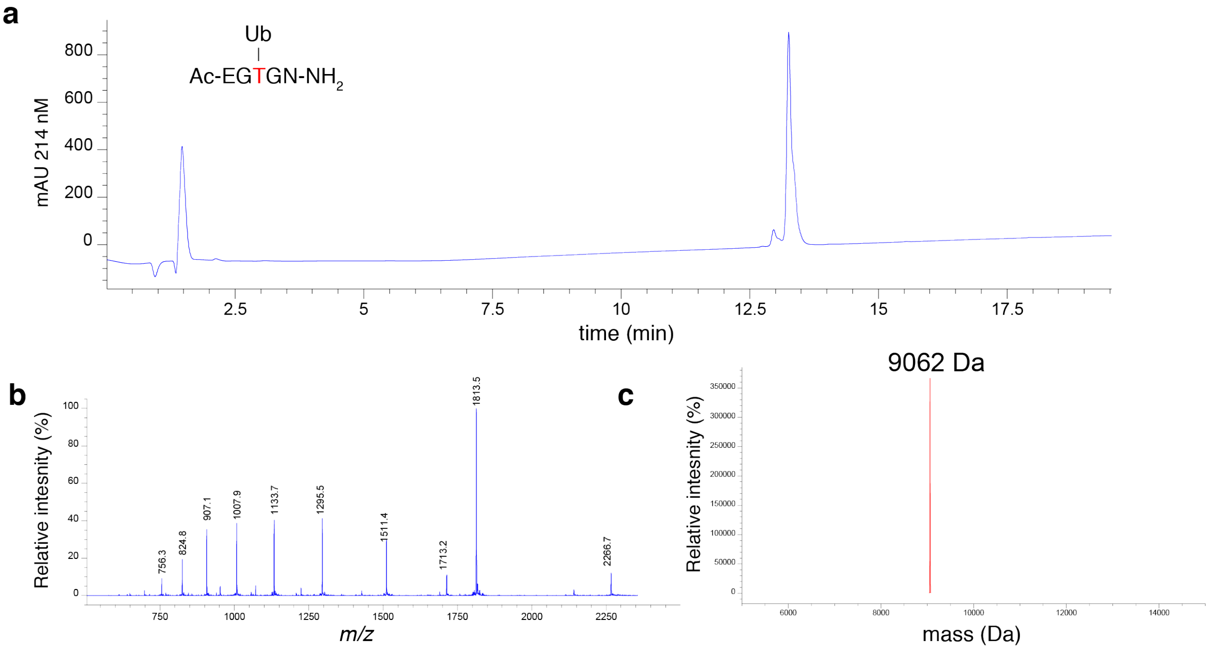

Figure S9. LC-MS characterization of purified Ub-EGTGN. (a) HPLC chromatogram for purified Ub-EGTGN monitoring at 214 nm. **(b)** Electrospray ionization mass spectrum for Ub-EGKGN. **(c)** Deconvoluted mass spectrum for Ub-EGTGN. Theoretical mass = 9064.45; observed mass = 9062 Da.

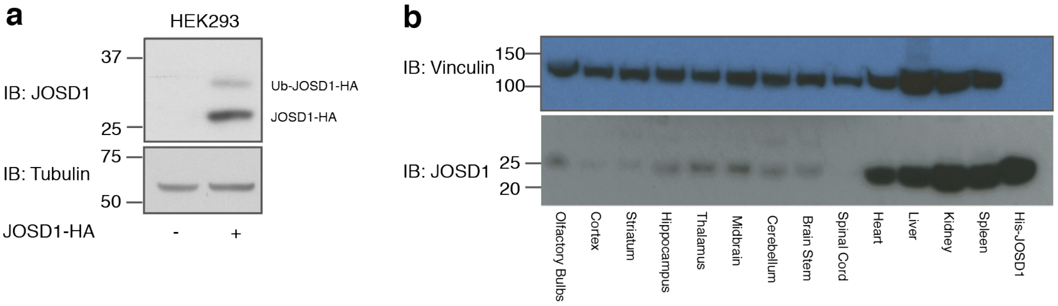

Figure S10. JOSD1 protein expression characterization: (a) JOSD1 immunoblot on cultured HEK293 cells. JOSD1 can be detected in HEK293 when transiently expressed. An upper band, corresponding to ubiquitinated JOSD1 is visible. **(b)** Protein lysates from the indicated tissues were immunoblotted with anti-JOSD1 antibody. JOSD1-His recombinantly expressed (30 ng) is used as positive control for accurate detection. JOSD1 is endogenously highly expressed in tissue as heart, liver, kidney and spleen while less abundant in brain regions.

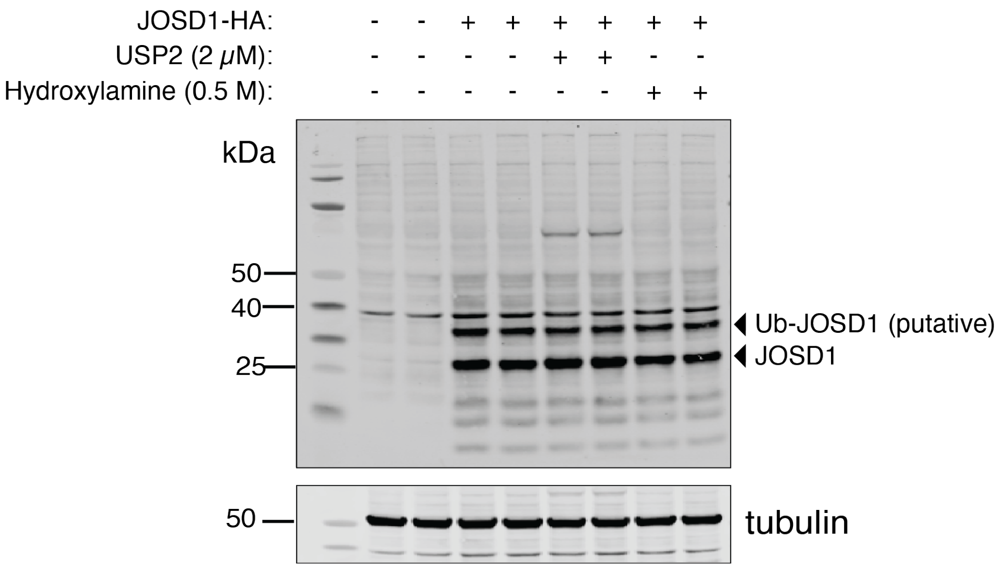

Figure S11. DUB and hydroxylamine resistance of modified JOSD1. HA-tagged JOSD1 was transiently overexpressed in HEK293 cells. We observed a higher molecular weight species which was presumed to be ubiquitinated as previously reported[44]. However, we could not independently confirm this. The higher molecular weight band was also stable after treatment of lysate with recombinant USP2 (2 μM) for 1 h at 37 °C, and, pH 9 0.5 M hydroxylamine for 0.5 h at 37 °C. This indicates that the Ub modification site is inaccessible by USP2 and is not ester-linked.

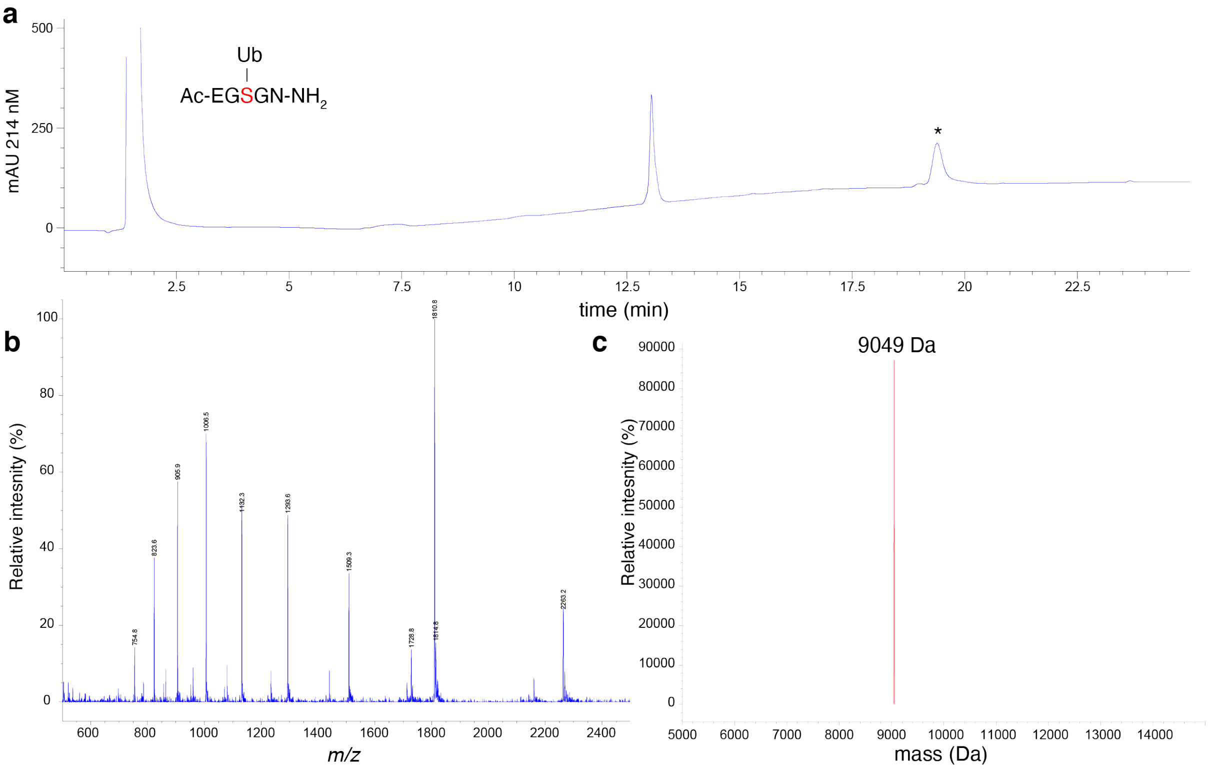

Figure S12. LC-MS characterization of purified Ub-EGSGN. (a) HPLC chromatogram for purified Ub-EGSGN monitoring at 214 nm. Asterisk corresponds to a polymer contaminant of the LC-MS instrument rather than the sample. **(b)** Electrospray ionization mass spectrum for

Ub-EGSGN. **(c)** Deconvoluted mass spectrum for Ub-EGSGN. Theoretical mass = 9050.29; observed mass = 9049 Da.

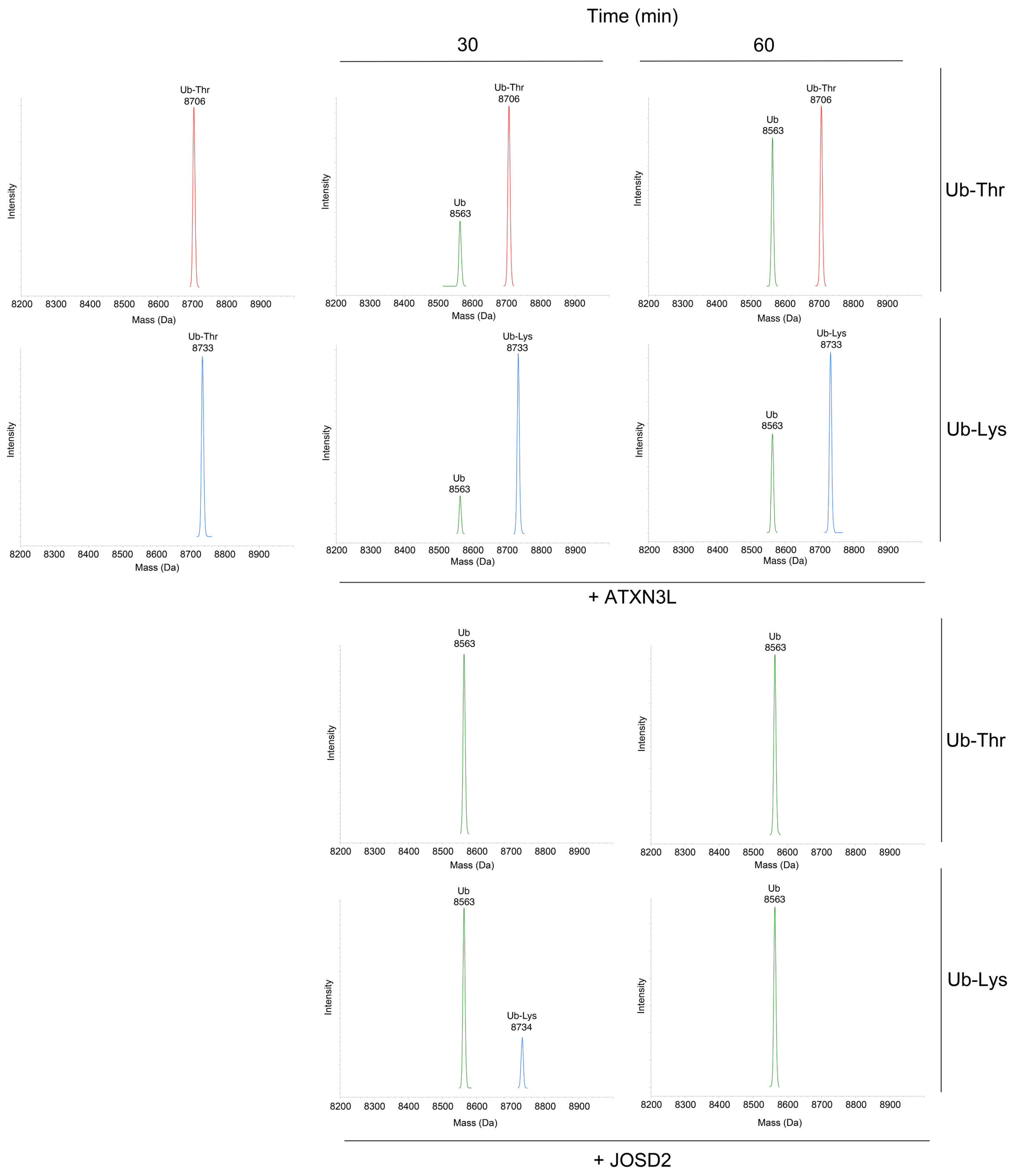

Figure S13. Complementary assessment of JOSD2 and ATXN3L activity towards Ub-Thr and Ub-Lys. DUBs (1.5 µM) and substrates (5 µM) were incubated for the indicated time point and reactions were quenched by the addition of trifluoroacetic acid. Reactions were then analysed by LC-MS. The deconvoluted electrospray mass spectrum for the peak corresponding to coeluting substrate (Ub-Thr or Ub-Lys) and product (Ub) are presented[5]. As ionisation characteristics of product and substrate would be similar, this provides a qualitative assessment of DUB activity. Theoretical mass of Ub-Lys = 8735.06; observed mass = 8733-

8734 Da. Theoretical mass of Ub-Thr = 8708.00observed mass = 8706 Da. Theoretical mass of Ub = 8564.84; observed mass = 8563 Da.

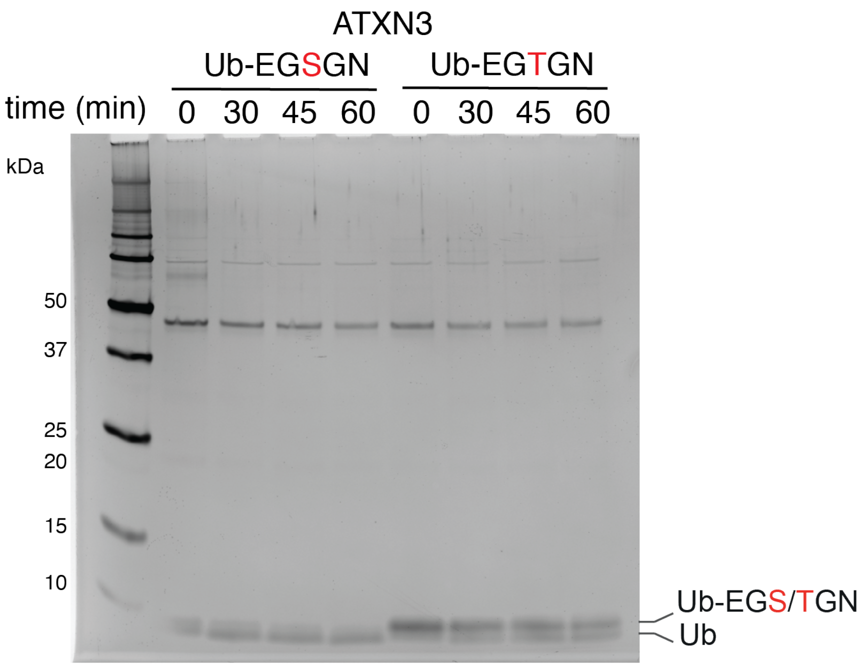

Figure S14. Assessment of ATXN3 selective activity towards the ubiquitinated serine and threonine peptides. ATXN3 (5 μM) was incubated with either Ub-EGSGN (5 μM) or Ub-EGTGN (5 μM) for the specified time. Reaction was resolved by SDS-PAGE and detection was carried out by silver staining.

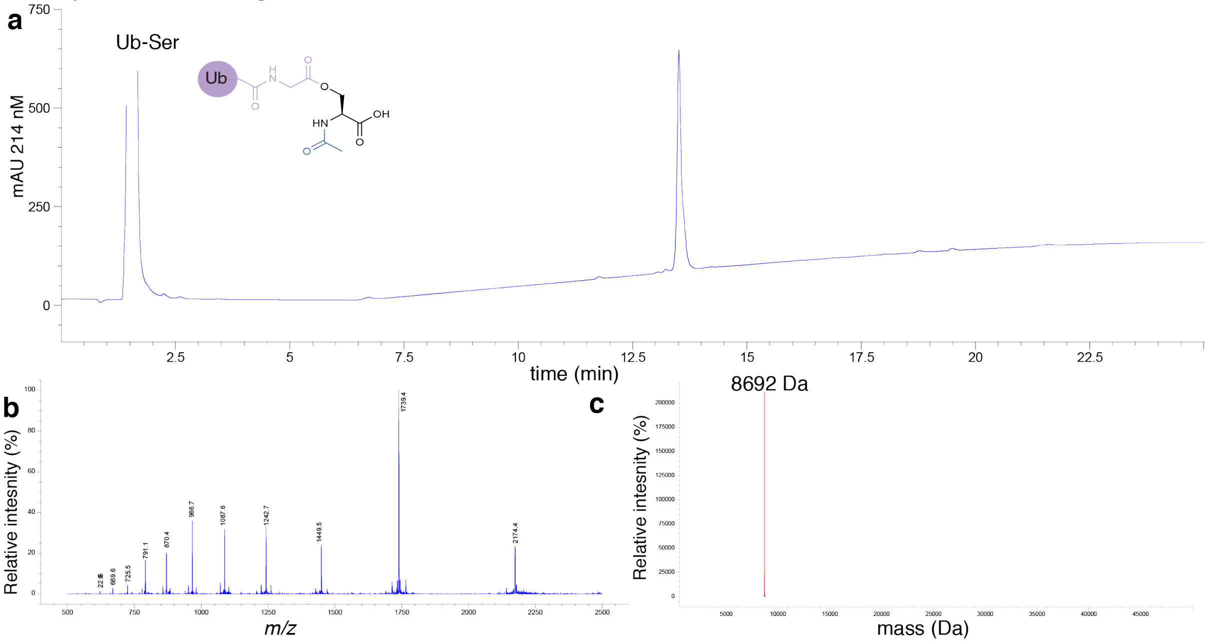

Figure S15. LC-MS characterization of purified Ub-Ser. (a) HPLC chromatogram for purified Ub-Ser monitoring at 214 nm. **(b)** Electrospray ionization mass spectrum for Ub-Ser. **(c)** Deconvoluted mass spectrum for Ub-Ser. Theoretical mass = 8694.13; observed mass = 8962 Da.

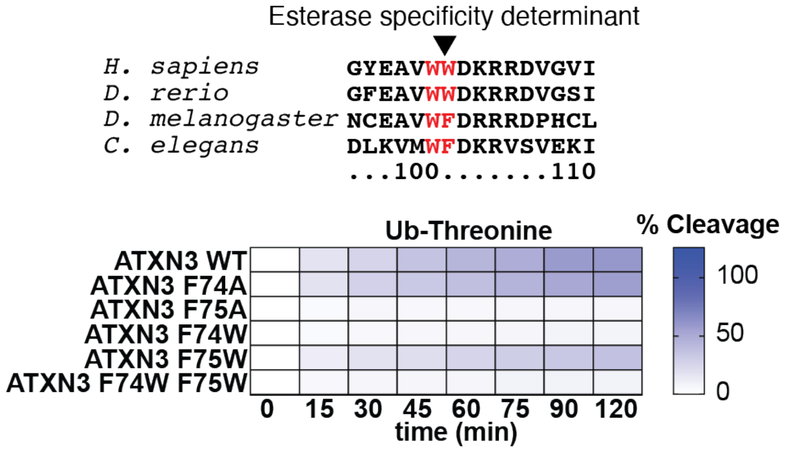

Figure S16. Sequence alignment of JOSD1 orthologues and mutational analysis of the esterase specificity determinant region in ATXN3. (Top) In some JOSD1 orthologues W101 is substituted for a phenylalanine residue. This is similar to ATXN3 and ATXN3L which have a phenylalanine residue at both W100 and W101 positions. (Bottom), Activity of ATXN3 mutants towards the Ub-Threonine substrate was assayed by MALDI-TOF mass spectrometry. Unlike JOSD1, the first hydrophobic residue (F74) is dispensable for esterase activity. However, the second F75 residue is required. F75 can also be substituted for tryptophan suggesting the phenylalanine and tryptophan residues found at the second position across the MJD class and their orthologues carry out a similar function.

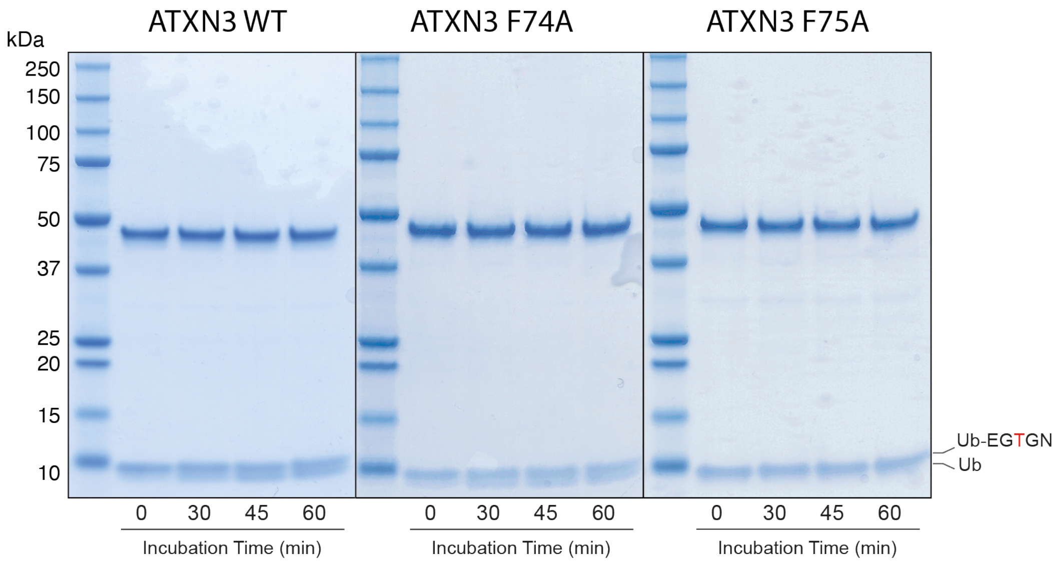

Figure S17. Assay of ATXN3 activity mutants towards ubiquitinated threonine peptide. As observed for Ub-Threonine substrate, activity towards the ubiquitinated threonine peptide substrate is only dependent on F75.

Enzyme	Known Substrate	Length	Tag	DU Number	Expression system	Purity	Average Mass	Concentration ng/µl	Concentration nM
USP1/UAF1	K63	FL	His	DU23056	bacteria	50%	90502	18.0	198.9
USP2	K63	FL	GST	DU13025	bacteria	50%	72731	7.5	103.1
USP4	K48	FL	His	DU14350	bacteria	40%	111006	7.2	64.9
USP5	K63	FL	His	DU15641	bacteria	90%	98880	0.2	1.8
USP6	K63	529-1406	GST	DU37745	insect	90%	125292	0.2	1.4
USP7	K11	FL	His	DU15644	bacteria	80%	130714	0.1	0.7
USP8	K63	FL	His	DU15645	bacteria	90%	130789	180.0	1376.4
USP9x	K11	1553-1995	GST	DU20628	bacteria	50%	78783	36.0	457.0
USP10	K48	FL	His	DU15647	bacteria	80%	91967	36.0	391.5
USP11	K48	FL	GST	DU20016	bacteria	20%	137621	12.0	87.2
USP12	K48	FL	His	DU24373	insect	40%	45127	12.0	265.9
USP15	K48	FL	GST	DU19772	bacteria	90%	137410	1.1	7.9
USP16	K63	FL	GST	DU46239	bacteria	80%	120394	3.6	29.9
USP20	K63	FL	GST	DU12807	bacteria	40%	128708	9.6	74.6
USP21	K48	196-565	His	DU22384	bacteria	80%	42852	7.2	168.0
USP25	K11	FL	-	DU21610	bacteria	80%	149037	1.8	12.1
USP27x	K11	FL	Dac	DU23206	insect	85%	76453	36.0	470.9
USP28	K11	FL	GST	DU20233	bacteria	60%	149310	3.6	24.1
USP30	K48	57-517	-	DU36294	bacteria	50%	52995	72.0	1358.8
USP36	K11	81-461	GST	DU4944	bacteria	80%	69387	27.0	389.2
USP45	K48	FL	GST	DU15681	bacteria	50%	118554	60.0	506.1
USP46	K48	FL	His	DU24347	insect	90%	44711	30.0	671.0
USP47	K48	FL	His	DU15682	insect	80%	159545	3.0	18.8
CYLD	K63	FL	His	DU1834	insect	90%	110553	72.0	651.3
OTULIN	M1	FL	-	DU43487	bacteria	95%	40462	0.02	0.4
OTU1	None	FL	GST	DU36559	bacteria	90%	65144	60.0	921.1
OTUD1	K63	270-481	His	DU25080	bacteria	90%	29718	0.2	6.1
OTUD3	K11	FL	His	DU21336	bacteria	80%	47566	3.6	75.7
OTUD5 p177S	K63	FL	-	DU21450	bacteria	70%	84826	90.0	1061.1
OTUD6A	None	FL	His	DU20889	bacteria	90%	35743	60.0	1678.8
OTUD6B	None	FL	GST	DU21140	bacteria	90%	64148	60.0	935.4
OTUB1	K48	FL	GST	DU19741	bacteria	90%	58106	1.8	31.0
OTUB2	K63	FL	GST	DU32795	bacteria	95%	54035	0.4	6.7
VCPIP1	K48	25-561	His	DU25040	bacteria	70%	137252	36.0	262.3
vOTU	K63	1 - 183	GST	DU45351	bacteria	>80%	47660	0.02	0.4
TRABID	K63	245-697	His	DU22468	bacteria	50%	54792	90.0	1642.7
A20	K48	1-366	-	DU32912	bacteria	90%	70843	9.0	127.1
Cezanne	K11	FL	GST	DU20899	bacteria	50%	119719	0.04	0.3
UCHL1	Ub-W	FL	His	DU15693	bacteria	95%	27267	0.3	11.0

UCHL3	Ub-W	FL	GST	DU21015	bacteria	80%	53984	0.03	0.6
UCHL5	Ub-W	FL	GST	DU12810	bacteria	80%	64300	210.0	3266.3
BAP1	Ub-W	FL	GST	DU12809	bacteria	70%	107182	120.0	1119.7
JOSD1	None	FL	His	DU20956	bacteria	95%	25641	60.0	2340.2
JOSD2	K48	FL	His	DU20941	bacteria	90%	23199	60.0	2586.6
ATXN3	K63	FL	GST	DU35448	bacteria	90%	69225	60.0	866.8
ATXN3L	K63	FL	His	DU23069	bacteria	70%	43191	60.0	1389.3
AMSH	K63	356-424	-	DU44746	bacteria	>80%	74769	180.0	2407.7
AMSH-LP	K63	265-436	-	DU15780	bacteria	80%	47266	0.1	1.9
MINDY1	K48 tetramer	1 - 469	MBP	DU59325	bacteria	>80%	96044	60.0	624.8
MINDY2	K48	241 - 504	GST	DU55390	bacteria	>80%	58182	60.0	1031.3
MINDY3	K48	FL	GST	DU47870	bacteria	>85%	76500	60.0	784.4
MINDY4	None	FL	GST	DU47893	bacteria	>20%	111100	60.0	540.1
ZUFSP	K63	231-578	GST	DU53621	bacteria	90%	66600	60.0	901.0
UAF1	None	FL	His	DU39055	insect	80%	81023	3.0	37.0

Table S1 Recombinant DUBS employed in this study. The known substrate corresponds to the substrate chosen in control experiments carried out to confirm DUB activity. If the DUB was expressed in truncated form then residue boundaries are stated (FL corresponds to full-length). DU number corresponds to MRC Reagents and Services internal reference number. Purity was approximated by Coomassie stained SDS-PAGE analysis. Concentration corresponds to that employed in MALDI-TOF assays unless otherwise stated.

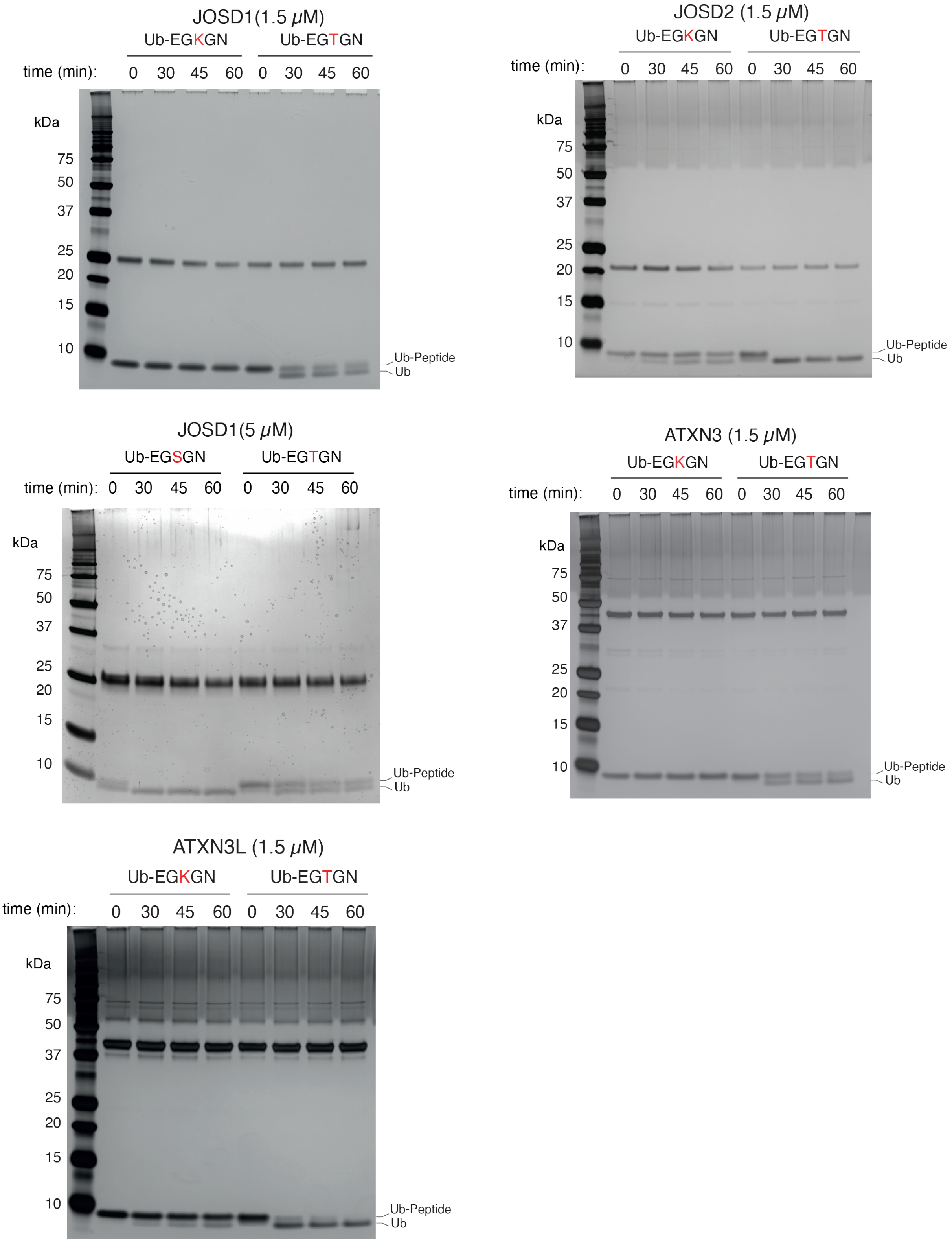

Full gels from Figure 4
